# CuCo‐Layered Double Hydroxide Nanosheets Grown on Hierarchical Carbonized Wood as Bifunctional Electrode for Supercapacitor and Hydrogen Evolution Reaction

**DOI:** 10.1002/advs.202508630

**Published:** 2025-09-08

**Authors:** Hewei Hou, Guiqing Lei, Huashuang Huo, Yuanyuan Yu, Zhenzhen Tang, Chengrong Qin, Douyong Min

**Affiliations:** ^1^ Guangxi Key Laboratory of Clean Pulp & Papermaking and Pollution Control School of Light Industry and Food Engineering Guangxi University Nanning 530004 China

**Keywords:** carbonized wood, CuCo‐LDH nanosheets, hydrogen evolution reaction, N/P co‐doping, supercapacitor

## Abstract

Carbonized wood has great potential as a self‐supported electrode for energy storage/conversion applications. However, developing efficient and economical bifunctional electrodes by customizing the surface structure remains a challenge. This study proposes a novel multifunctional electrode design strategy, using N/P co‐doped carbonized wood (NPCW) as carriers and in situ grows copper nanoparticles (Cu NPs) as nucleation centers to induce vertical growth of CuCo‐layered double hydroxid (LDH) nanosheets along the substrate. This method avoids the disordered stacking of catalysts and forms the “carbon‐metal‐LDH” tertiary conductive network. Therefore, the hierarchical CuCo‐LDH@Cu/NPCW is successfully fabricated. Benefiting from the hierarchical ultrathin nanosheet arrays and the strong electronic interactions between CuCo‐LDH and Cu/NPCW substrates, CuCo‐LDH@Cu/NPCW exhibits a high specific capacitance of 26.24 F cm^−2^ at 2 mA cm^−2^, with a capacitance retention of 96.70% after 10 000 cycling tests. The assembled symmetric supercapacitor (SSC) achieves a high energy density of 0.80 mWh cm^−2^ at 7.50 mW cm^−2^. In addition, CuCo‐LDH@Cu/NPCW exhibits excellent HER performance with high activity (η_10_ = 32 mV), low Tafel slope (78 mV dec^−1^), and excellent long‐term stability. This work realizes the controllable preparation of high‐performance bifunctional electrodes and provides new ideas for the application of biomass‐derived materials in energy storage and conversion.

## Graphical abstract

The N/P co‐doped carbonized wood (NPCW) with open channels is used as the carrier, and in situ uniformly grown copper nanoparticles (Cu NPs) as nucleation centers to induce the vertical growth of CuCo‐LDH nanosheets along the substrate, forming a “carbon‐metal‐LDH” tertiary conductive network, which realizes the synergistic enhancement of high‐performance energy storage and electrocatalytic performance of HER.

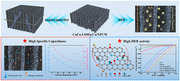

## 1 Introduction

The development of highly efficient, stable, and low‐cost energy storage and conversion technologies has become a major focus of scientific research and industrial applications, driven by the global energy structure transition and the “carbon neutralization” goal.^[^ ^1^, ^2^ ^]^ Among the applications of energy conversion and storage technology, supercapacitors (SCs) have demonstrated irreplaceability in high‐power scenarios such as smart grids and rail transportation by the advantages of high density, long cycle life, and fast charging speed.^[^ ^3^, ^4^ ^]^ In addition, hydrogen is considered the most promising energy source to replace traditional fossil fuels due to its high calorific value and zero carbon emissions. The electrocatalytic hydrogen evolution reaction (HER) shows attractive application prospects as the most ideal way to produce hydrogen.^[^ ^5^, ^6^ ^]^ However, the existing electrode materials face significant challenges for applications in the field of energy storage and conversion: 1) The carbon‐based materials for supercapacitors (e.g., activated carbon, graphene) are limited by the double‐electric‐layer energy storage mechanism, and the energy densities are generally low, which makes it difficult to fulfill the practical demands^[^ ^7^, ^8^, ^9^, ^10^ ^]^; 2) HER catalysts rely heavily on platinum group precious metals, but their high cost and low reserves constrain industrial applications;^[^ ^11^ ^]^ 3) The design of traditional electrode materials focuses on the optimization of the single performance, and lacks the exploration of the synergistic mechanism of the dual function of energy storage and conversion. Therefore, the construction of a multifunctional electrode material with high specific capacity, excellent catalytic activity, and high cycle life has become an important direction to break through the current technical bottleneck.

Wood is an abundant lignocellulosic material with a unique open channel structure, multiple porous structures, and excellent mechanical properties, which are maintained after carbonization, making it an excellent precursor for use as a self‐supported electrode carrier.^[^ ^12^, ^13^ ^]^ However, the carbonized wood (CW) directly used as a self‐supported electrode for energy storage and electrocatalysis does not exhibit desirable electrochemical properties.^[^ ^14^ ^]^ It has been reported that N/P co‐doping can modulate the pore structure and surface properties of CW, improving its capacitive properties and electrocatalytic activity.^[^ ^15^, ^16^ ^]^ For example, Wang et al.^[^ ^17^ ^]^ prepared a phosphorus‐doped wood‐derived carbon electrode by phytate treatment, which significantly enhanced the capacitive performance of the material. Li et al.^[^ ^18^ ^]^ constructed an integrated carbon electrode with a porous structure by encapsulating nickel nanoparticles in N‐doped carbonized wood framework (Ni@NCW), which exhibited excellent HER performance. Therefore, the development of high‐performance multifunctional electrode materials for energy storage and conversion by using N/P co‐doping carbonized wood as the carrier has a broad prospect.

At present, research is underway to develop multifunctional electrode materials, which include transition metal oxides, metal sulfides, phosphides, and layered double hydroxides (LDHs).^[^ ^19^, ^20^, ^21^ ^]^ LDHs are widely used in supercapacitors and electrocatalytic hydrogen evolution because of the excellent layered structure, large specific surface, high theoretical specific capacitance, and abundant active sites.^[^ ^22^, ^23^ ^]^ However, the low conductivity and instability of the interlayer structure limit the comprehensive performance in practical applications. The construction of binary transition metal hydroxides can maximize the synergistic effect of interlayer metal cations and further improve the electrochemical performance and structural stability while maintaining the advantages of LDH.^[^ ^24^ ^]^ Among many transition metal hydroxides, Cu‐Co hydroxide is a potential candidate due to the advantages of environmental friendliness, low cost, and various oxidation valence states. Xu et al.^[^ ^25^ ^]^ synthesized hierarchical hollow structures of nitrogen‐doped graphene quantum dots (NGQDs) embedded in CuCo‐LDH by hydrothermal and impregnation methods, which significantly enhanced the energy storage capacity of the materials. Wang et al.^[^ ^26^ ^]^ successfully synthesized oxygen vacancy‐rich, amorphous/crystalline low ruthenium‐doped CoCu‐LDH on nickel foam by using the self‐sacrificial template method and chemical etching synthesis strategy, which exhibited efficient hydrolysis. Although CuCo‐LDH has shown excellent capacitance performance and HER performance, it still suffers from poor intrinsic conductivity, collapse‐prone interlayer structures, non‐uniform growth, and susceptibility to disordered stacking in the application process. Therefore, the introduction of new components to solve the bottlenecks of LDH and further elucidate its mechanism remains a major challenge.

Herein, we present a method to induce the vertical growth of CuCo‐LDH nanosheets along the substrate by using N/P co‐doped carbonized wood as the carrier and uniformly grown copper nanoparticles (Cu NPs) as the growth sites. The method avoids the disordered stacking of LDH nanosheets, and the prepared self‐supported electrodes exhibit neatly aligned open channels, hierarchical pore structures, and uniformly loaded ultrathin nanosheet structures. In addition, uniformly grown nanosheet arrays have abundant active sites, which enhance intrinsic activity, and form strong electronic interactions with the Cu/NPCW substrate, which enhances electrical conductivity and structural stability, thus significantly improving capacitance and catalytic performance. CuCo‐LDH@Cu/NPCW exhibits excellent performance as supercapacitor electrodes with a specific capacitance of 26.24 F cm^−2^ (749.71 F g^−1^) at a current density of 2 mA cm^−2^. The assembled symmetric supercapacitor (SSC) demonstrates a high energy density of 0.80 mWh cm^−2^ at a power density of 7.50 mW cm^−2^. In addition, CuCo‐LDH@Cu/NPCW exhibits an excellent HER catalytic activity, with low overpotentials of 32 and 166 mV at current densities of 10 and 100 mA cm^−2^, respectively. The in‐situ electrochemical impedance spectroscopy reveals that the synergistic interaction of NPCW and CuCo‐LDH improves the reaction kinetics of the HER process. The findings in this work provide a new idea for the application of biomass‐derived carbon in energy storage and conversion, simultaneously.

## 2 Results and Discussion

### 2.1 Synthesis and Characterization

**Figure 1:**
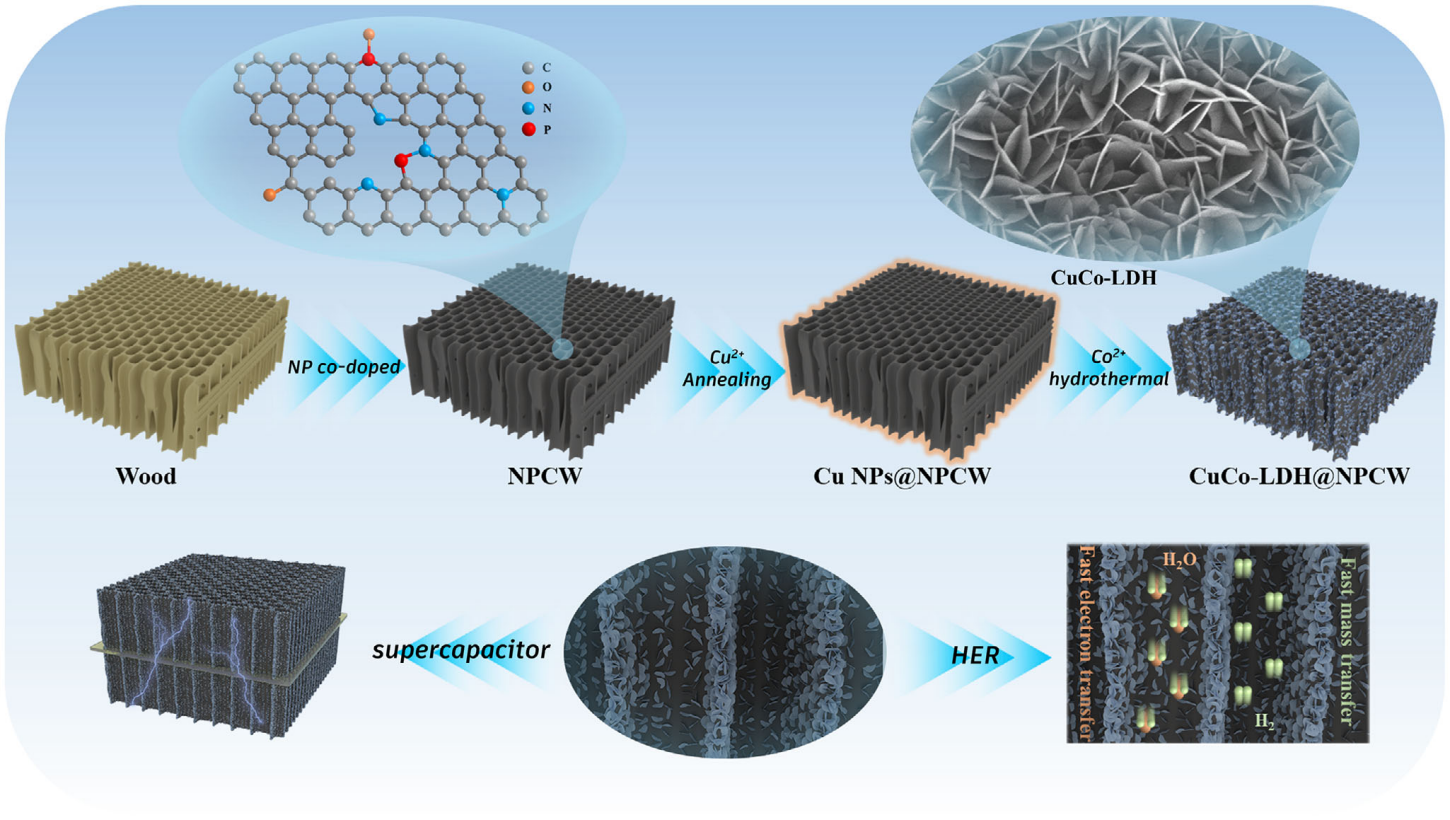
Schematic illustration of the synthesis of CuCo‐LDH@Cu/NPCW.

The schematic synthesis of the self‐supported CuCo‐LDH@Cu/NPCW electrode is shown in **Figure** **1**. First, the softwood (pine wood) (Figure S1a, Supporting Information) was pre‐oxidized at 220 °C in an air atmosphere to obtain pre‐oxidized wood (Figure S1b, Supporting Information). Subsequently, NPCW (T. Ma, H. Lin, B. Jia. Figure S1e, Supporting Information) was obtained by carbonizing the pre‐oxidized wood at 1000 °C with NH_4_H_2_PO_4_ as the dopant in a nitrogen atmosphere. The dimensional shrinkage of NPCW was significantly reduced during the carbonizing process as compared with that of CW due to the fact that the doping of N and P elements stabilizes the structure to a certain extent (Figure S1c, e, Supporting Information). Then, NPCW was vacuum impregnated with CuCl_2_, which was annealed to obtain Cu NPs@NPCW (Figure S1f, Supporting Information). Finally, CuCo‐LDH nanosheets were uniformly in situ grown by hydrothermal reaction using Cu NPs as nucleation centers to obtain CuCo‐LDH@Cu/NPCW (Figure S1g, Supporting Information).

**Figure 2:**
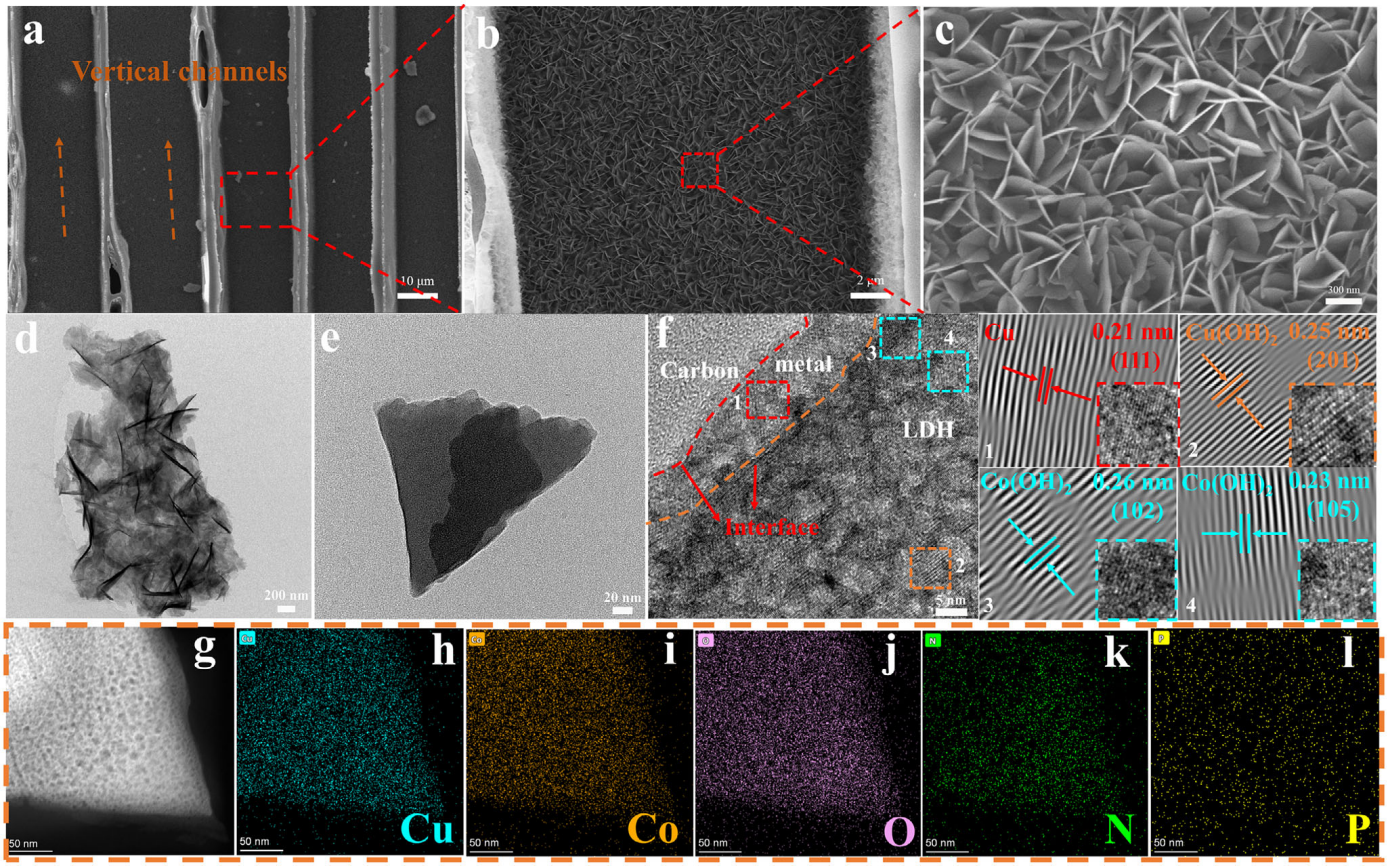
a–c) SEM images of CuCo‐LDH@Cu/NPCW, longitudinal section. d,e) TEM and f) HRTEM images of CuCo‐LDH@Cu/NPCW. g) HAADF‐STEM images and h–l) associated elemental mapping images of CuCo‐LDH@Cu /NPCW.

Notably, the porous structure of NPCW was shown in Figure S2 (Supporting Information). It can be observed from the cross‐sectional scanning electron microscopy (SEM) images that the fiber tracheids with diameters of 10–30 µm form a 3D porous structure (Figure S2a, Supporting Information). In the longitudinal section, vertical open channels with low tortuosity can be observed (Figure S2b, Supporting Information), which can provide excellent transport channels for the electrolyte and effective energy storage space.^[^ ^27^, ^28^ ^]^ In addition, microchannels formed by ray parenchyma cells (Figure S2b, Supporting Information) and a large number of nanopores etched out during the doping process (Figure S2c, Supporting Information) shortened the lateral diffusion distance of the electrolyte, facilitating the rapid transport of ions and the release of gas bubbles.^[^ ^29^ ^]^ These structures also significantly increased the specific surface area of the substrate material, providing more active sites and favoring the uniform loading of the catalyst.^[^ ^30^ ^]^ After vacuum impregnation and annealing, Cu NPs with an average size of 51.94 nm were uniformly in situ grown in the open channels of NPCW (Figure S3a,b, Supporting Information),^[^ ^15^ ^]^ which can enhance its electrical conductivity, accelerate the electron transfer, and provide the sites for the subsequent formation of CuCo‐LDH nanosheets. The CuCo‐LDH nanosheets were uniformly loaded on the surface and in the open channels of NPCW to form the 3D network structure (**Figure** **2**b,c; S4b, Supporting Information). The ultrathin CuCo‐LDH nanosheet structures have an increased active surface, which can provide more active sites and edge defects, significantly improving the capacitance and catalytic activity of the materials. Interestingly, the uniform and dense dispersion of CuCo‐LDH on the carbonized wood greatly preserved the channels for the electrolyte diffusion and bubble escape (Figures 2a; S4a, Supporting Information), effectively increasing the contact area and accessibility of the electrode to the electrolyte, and thus facilitating the kinetic process of the redox reaction.^[^ ^31^ ^]^ However, the dense and uniform CuCo‐LDH was not observed from CuCo‐LDH@Cu/CW (Figure S3c,d, Supporting Information). The SEM‐EDS spectra of CuCo‐LDH@Cu/NPCW (Figure S5a–g, Supporting Information) demonstrated that carbon (C), oxygen (O), nitrogen (N), phosphorus (P), cobalt (Co), and copper (Cu) elements were uniformly distributed on the carbonized wood, which confirmed that CuCo‐LDH had been successfully loaded on NPCW. To further confirm the metal loading, the content of Cu and Co elements determined by inductively coupled plasma optical emission spectroscopy (ICP‐OES) is demonstrated in Table S1 (Supporting Information).

Figure 2d,e demonstrates the ultrathin nanosheet structure of CuCo‐LDH@Cu/ NPCW, which can increase the active sites and promote electron transfer and electrolyte transport, improving the electrocatalytic activity.^[^ ^25^ ^]^ Figure 2f shows the lattice fringes with spacings of 0.21, 0.25, 0.26, and 0.23 nm, attributing to the (111) plane of Cu, (201) plane of Cu(OH)_2_, (102) plane, and (105) plane of Co(OH)_2_, respectively.^[^ ^32^ ^]^ Significantly, the lattice stripes of Cu were observed, indicating that Cu NPs were not completely transformed into Cu(OH)_2_, and the residue Cu NPs acted as a bridge between NPCW and CuCo‐LDH to form a “carbon‐metal‐LDH” tertiary conductive network, which can enhance the charge transfer rate and thus improve the electrochemical performance. Figure S6 (Supporting Information) indicates the presence of Cu, Cu(OH)_2_, and Co(OH)_2_ through the (111), (231), and (105) planes, confirming the crystallinity of CuCo‐LDH. Figure 2g–i illustrates the uniform distribution of C, O, N, P, Co, and Cu elements in the CuCo‐LDH@Cu/NPCW electrode. The percentages of C, O, N, P, Cu, and Co were calculated as 51.70%, 30.03%, 4.34%, 1.75%, 4.94% and 7.24%, respectively (Figure S7, Supporting Information). The results revealed the formation of CuCo‐LDH nanosheets in CuCo‐LDH@Cu/NPCW.

**Figure 3:**
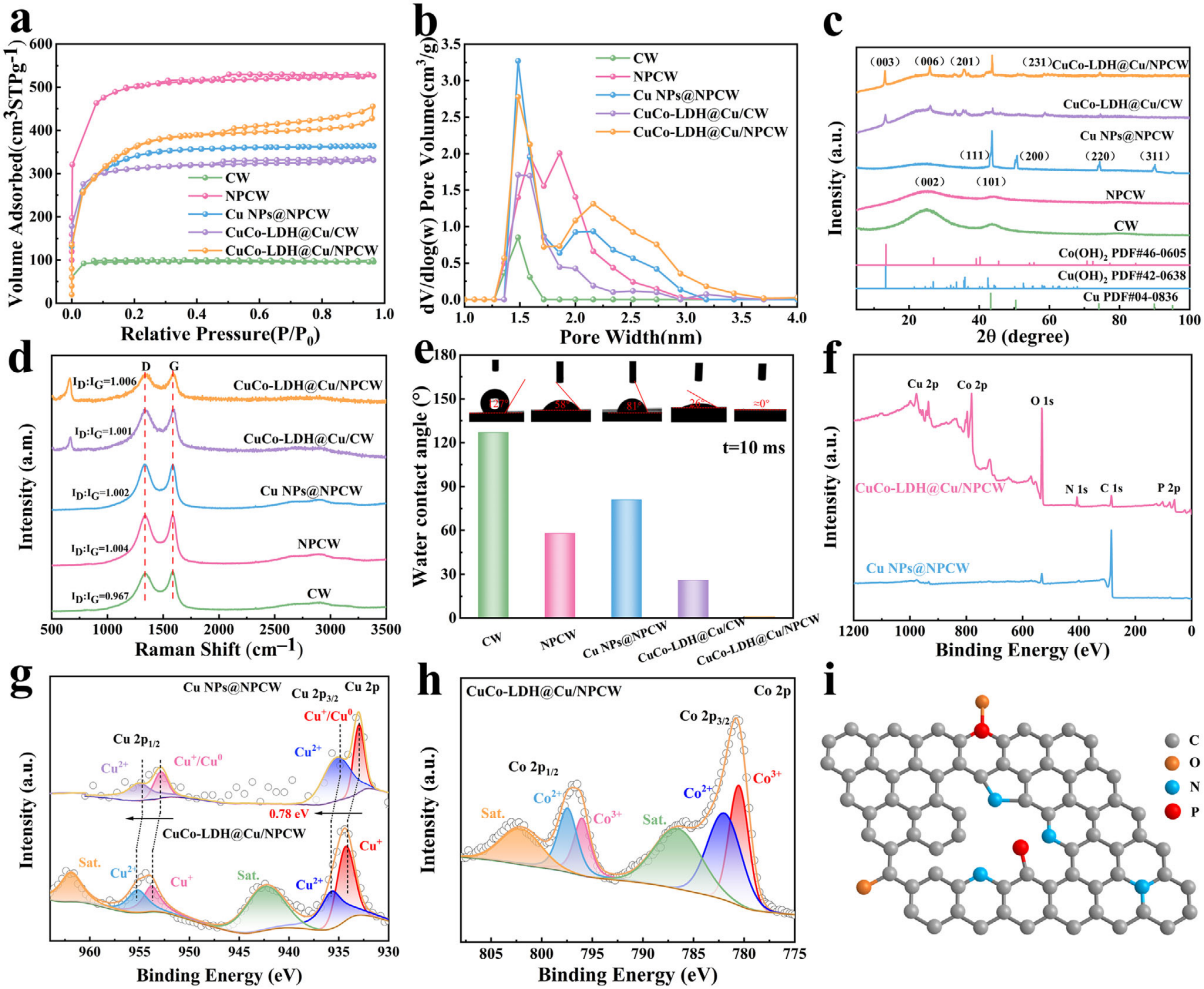
Physical characterization of electrodes. a) N_2_ adsorption and resolution isotherms, and b) pore size distribution; c) XRD spectra; d) Raman spectra; e) hydrophilic contact angles; f) XPS survey spectrum of Cu NPs@NPCW, and CuCo‐LDH@Cu/NPCW electrodes; High‐resolution XPS spectra of samples for g) Cu 2p h) Co 2p; i) Schematic illustration of N /P/O bonds in the carbon skeleton.

As shown in **Figure** **3**a, all the samples showed type IV isothermal curves, suggesting a large number of micropores and mesopores in the samples.^[^ ^33^ ^]^ CuCo‐LDH@Cu/NPCW had the largest pore volume of 0.46 cm^3^ g^−1^, which mainly consisted of micropores and mesopores with the most obvious hierarchical porous structure (Figures 3b; S8, Supporting Information),^[^ ^34^ ^]^ which can facilitate the dynamic exchange of electrolyte ions and the escape of gas during the HER process.^[^ ^35^ ^]^ Notably, due to the etching effect of N, P co‐doping, the specific surface area and microporous volume of NPCW were significantly larger than those of CW. For example, the specific surface area and average pore size of NPCW were 1678.72 m^2^ g^−1^ and 0.49 nm, respectively (Table S2, Supporting Information). However, the specific surface area of Cu NPs@NPCW was reduced to 1223.38 m^2^ g^−1^, while the average pore size was increased to 0.67 nm, which can be attributed to the partial blockage of micropores by Cu NPs. With the introduction of CuCo‐LDH nanosheets, the specific surface area of CuCo‐LDH@Cu/NPCW was increased to 1419.22 m^2^ g^−1^ while the average pore size was decreased to 0.63 nm, attributing to the uniform growth of nanosheets, which can improve the electrochemical activity of the electrode.^[^ ^24^ ^]^

Figure 3c illustrates that CW and NPCW possessed the typical disordered structures at 24° and 43°, corresponding to the (002) and (101) diffractions of graphitic carbon.^[^ ^36^ ^]^ Compared to CW, a weak characteristic peak and a downward shift were observed from NPCW, attributing to the N/P co‐doping, which decreased the graphitization, while increasing the lattice constants.^[^ ^37^ ^]^ Cu NPs@NPCW exhibited five characteristic peaks at 43.30°, 50.40°, 74.10°, 89.90°, and 95.10°, which were consistent with the copper phase (JCPDS No. 04–0836), confirming the growth of Cu NPs.^[^ ^15^ ^]^ The sharp peak at 13.16° corresponding to the (003) plane of LDH was observed from CuCo‐LDH@Cu/CW and CuCo‐LDH@Cu/NPCW, demonstrating the formation of LDH.^[^ ^38^ ^]^ The peaks at 21.51°, 27.01°, 35.91°, 36.81°, 40.42°, and 58.68° were attributed to the (110), (006), (201), (211), (102), and (231) planes of Cu(OH)_2_ (JCPDS# 42–0638) and Co(OH)_2_ (JCPDS# 46–0605), respectively, demonstrating the formation of CuCo‐LDH.^[^ ^32^ ^]^ The characteristic peaks at 43.30° and 74.10° assigned to the Cu phase were also observed from CuCo‐LDH@Cu/CW and CuCo‐LDH@Cu/NPCW, confirming the presence of Cu. The diffraction peak intensity of CuCo‐LDH gradually increased while the diffraction peak intensity of Cu gradually decreased with the increase of Co(NO_3_)_2_, which was attributed to the gradual growth of CuCo‐LDH with Cu NPs as the nucleation centers (Figure S9, Supporting Information). Figure 3d shows that all the samples exhibited two distinct bands near 1343 and 1598 cm^−1^, corresponding to the disordered carbon (D band) and graphitic carbon (G band), respectively.^[^ ^18^ ^]^ The I_D_/I_G_ ratios were calculated as 0.9670, 1.004, 1.002, 1.001, and 1.006 for CW, NPCW, Cu NPs@NPCW, CuCo‐LDH@Cu/CW, and CuCo‐LDH@Cu/NPCW, respectively, suggesting that the etching of NH_3_ and phosphoric acid during NP co‐doping, as well as in‐situ growth of CuCo‐LDH nanosheets, promoted the growth of defects.^[^ ^39^ ^]^ Figure S10 (Supporting Information) shows that the characteristic vibrational peaks at 1036.60, 720.70, 515.50, 469.50, and 184.60 cm^−1^ were attributed to the CuCo‐LDH, which further confirmed the growth of CuCo‐LDH nanosheets.^[^ ^40^ ^]^ Figure 3e shows that the hydrophilic contact angles of CW, NPCW, Cu NPs@NPCW, CuCo‐LDH@Cu/CW, and CuCo‐LDH@Cu/NPCW were 127°, 58°, 81°, 26°, and 0°, respectively. The improved hydrophilicity can optimize the wettability and mass transfer at the electrode‐electrolyte interface, which significantly improves the capacitance performance and enhances the reaction kinetics of HER.^[^ ^41^ ^]^

Figure 3f confirms the presence of the Cu, Co, C, O, N, and P elements. Figure 3g shows that CuCo‐LDH@ Cu/NPCW had two characteristic peaks at 934.30 and 935.80 eV, assigned to Cu 2*p*_3/2_, and other characteristic peaks at 953.80 and 955.20 eV, assigned to Cu 2*p*_1/2_, and the satellite peaks at 953.80 and 955.20 eV. Compared to Cu NPs@NPCW, the positive shift was observed from Cu 2*p*_3/2_ and Cu 2*p*_1/2_ peaks. The result can be explained by the increasing binding energy, which was attributed to the oxidation of Cu^0^ to Cu^2+^(Figure 3g).^[^ ^25^ ^]^ As for Co 2*p*, the characteristic peaks at 780.50 and 781.90 eV were assigned to Co 2*p*_3/2_, and other characteristic peaks at 796.10 and 797.47 eV were assigned to Co 2*p*_1/2_, and two satellite peaks at 786.50 and 802.10 eV were observed (Figure 3h).^[^ ^25^, ^38^ ^]^ As for C 1*s*, the peaks at 284.60, 285.70, 287.40, and 289.20 eV were assigned to C═C (sp^2^), C─N/C─P (sp^3^), C─O, and C═O/C═N, respectively.^[^ ^42^ ^]^ However, the peak at 289.20 eV assigned to C═O/C═N was not observed from Cu NPs@NPCW (Figure S11a, Supporting Information). As for O 1s, the characteristic peaks at 531.10, 532.30, and 533.20 eV were assigned to the C═O, C─O, and O═C─O─ bonds, respectively (Figure S11b, Supporting Information).^[^ ^43^ ^]^ In addition, oxygen‐containing functional groups, especially carboxyl and carbonyl groups, could enhance the hydrophilicity of the materials, introduce pseudocapacitance to improve capacitance performance, and further improve electrochemical activity. As for N 1*s*, the characteristic peaks at 397.90, 398.80, 399.80, 401.10, and 402.10 eV were assigned to pyridine nitrogen, metal nitrogen (M─N), pyrrole nitrogen, graphitic nitrogen, and nitrogen oxide, respectively(Figure S11c, Supporting Information).^[^ ^15^ ^]^ The presence of M─N bonds indicated the metal was successfully loaded through the M─N─C bond of the carbon substrate, which can modulate the electronic structure of the metal, thereby enhancing HER activity and capacitive performance of the electrode. In addition, pyrrole‐N was an electron donor that can contribute to the pseudocapacitance and enhance the catalytic activity and stability of the electrode, whereas pyridine‐N can introduce active sites and optimize H* adsorption, and graphite‐N can facilitate charge transport. Nitrogen (N) mainly exhibits electropositive properties in carbon substrates and can enhance the conductivity of carbon substrates by providing electrons.^[^ ^42^, ^44^ ^]^ As for P 2*p*, the peaks at 132.40 and 134.30 eV were assigned to P─C and P─O, respectively (Figure S11d, Supporting Information). The doping of P elements formed distorted P─C and P─O bonds, creating strain defects and vacancies in the carbon matrix, which can enhance the pseudocapacitive activity and regulate the electronic structure of metals, improve the hydrophilicity and conductivity, thereby enhancing the accessibility of the reactants to the active sites and effectively promoting the catalytic reaction.^[^ ^43^, ^45^ ^]^ The N/P/O bond configuration in the carbon skeleton was schematically proposed in Figure 3i.^[^ ^46^, ^47^ ^]^

### 2.2 Supercapacitive Property

**Figure 4:**
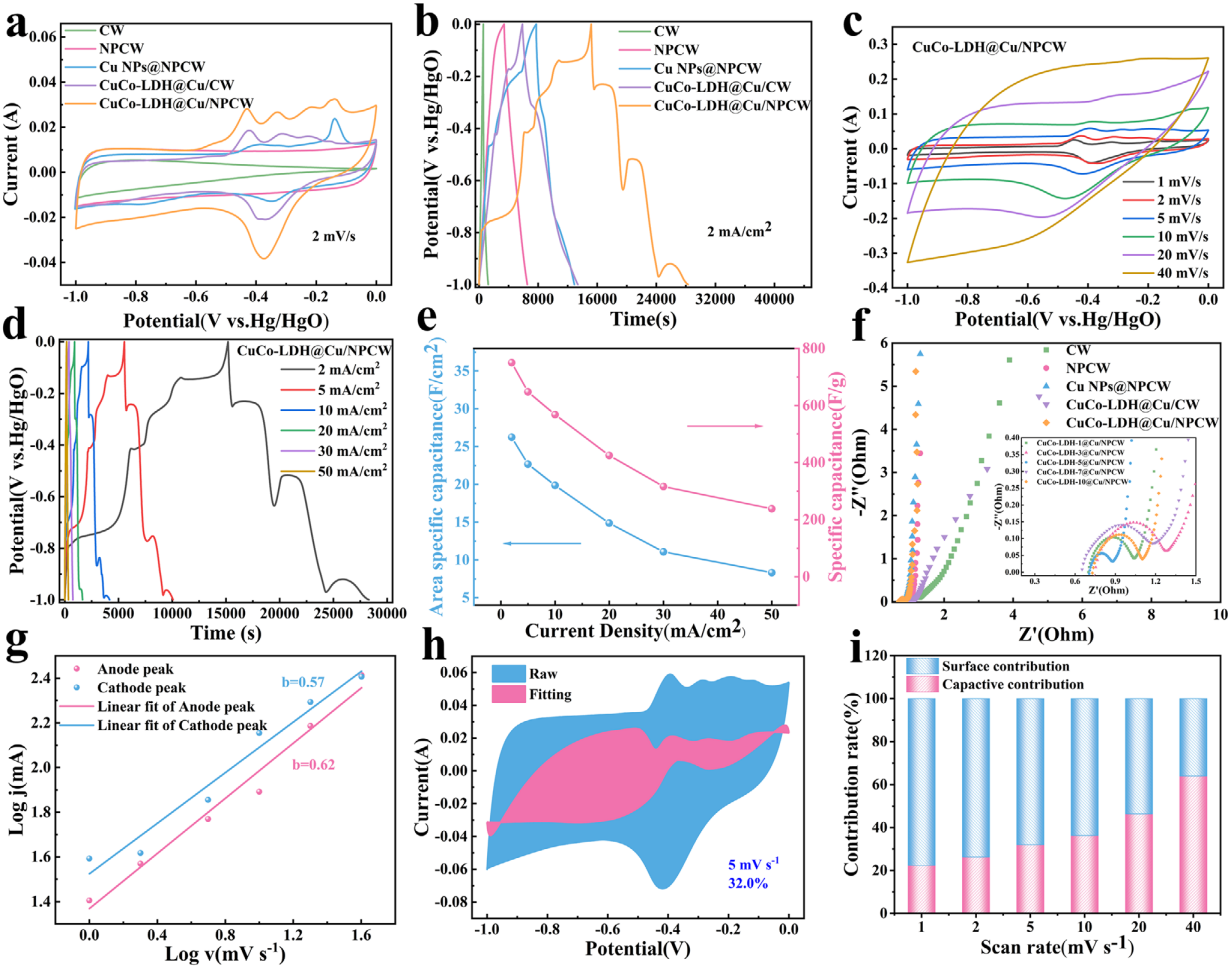
The Supercapacitor performance of electrodes: a) CV curves at 2 mV s^−1^; b) GCD curves at 2 mA cm^−2^; c) CV curves of CuCo‐LDH@Cu/NPCW electrode at 1 to 40 mV s^−1^; d) GCD curves of CuCo‐LDH@Cu/NPCW electrode at 2 to 50 mA cm^−2^; e) Specific capacitance of CuCo‐LDH@Cu/NPCW electrode; f) EIS spectra; g) Linear relation graph of log(i) and log(v) of CV curves; h) The ratio of capacitance contribution of CuCo‐LDH@Cu/NPCW electrode to total capacitance at scanning rates of 5 mV s^−1^; i) Percentage of capacitance and diffusion control contribution of the Co‐LDH@Cu/NPCW electrode.

As shown in **Figure** **4**a, the CV curves of CW, NPCW, Cu NPs@NPCW, CuCo‐LDH@Cu/CW, and CuCo‐LDH@Cu/NPCW were obtained at a scanning rate of 2 mV⋅s^−1^. The N/P co‐doped electrodes showed rectangular cyclic voltammetry curves, indicating the excellent capacitive properties.^[^ ^1^ ^]^ Comparing with Cu NPs@NPCW (Figure S12a, Supporting Information), CuCo‐LDH@Cu/NPCW shows more intense redox peaks, suggesting the presence of a rapid redox reaction (Figure S13a, Supporting Information). The obvious redox peaks in the CV curves can be rationalized by the following reactions:^[^ ^25^, ^48^ ^]^

(1) $$2Cu+2OH^{-}↔Cu_{2}O+H_{2}O+2e^{-}$$

(2) $$Cu_{2}O+2OH^{-}\;↔2CuO+H_{2}\;O+2e^{-}$$

(3) $$Cu{\left(OH\right)}_{2}+OH^{-}\;↔CuOOH+H_{2}\;O+e^{-}$$

(4) $$CuOOH+OH^{-}\;↔Cu_{2}O+H_{2}\;O+e^{-}$$

(5) $$Co{\left(OH\right)}_{2}+OH^{-}↔CoOOH+H_{2}\;O+e^{-}$$

(6) $$CoOOH+OH^{-}\;↔CoO_{2}+H_{2}\;O+e^{-}$$

In addition, the CuCo‐LDH@Cu/NPCW electrode had the largest integrated area of the CV curve, indicating it had the best capacitive performance.^[^ ^49^ ^]^ Figure 4b illustrates the corresponding GCD curves obtained for the electrodes at 2 mA cm^−2^, which provided a visual comparison of the discharge times of the electrodes. Among them, CuCo‐LDH@Cu/ NPCW had the longest discharge time, indicating a stronger Faraday redox reaction, higher electrochemical activity, and the largest specific capacitance (Figure S14, Supporting Information), which was consistent with the results of CV analysis.^[^ ^12^, ^50^ ^]^ The excellent capacitance performance may be attributed to the uniform channels, hierarchical porous structure, high hydrophilicity, and N/P active sites provided by NPCW; and the high surface area, interlayer structure, and bimetallic synergistic pseudocapacitance provided by the uniformly grown CuCo‐LDH nanosheets. As shown in Figure 4c, with the increasing scanning rate in the voltage range of ‐1–0 V, the redox peaks of the CuCo‐LDH@Cu/NPCW gradually moved to both sides of the voltage window, which was mainly due to charge diffusion polarization within the electrode.^[^ ^25^, ^51^ ^]^ Figure 4d displays the GCD curves of CuCo‐LDH@Cu/NPCW at current densities ranging from 2 to 50 mA cm^−2^. The obvious charging and discharging plateaus can be seen from the GCD curves, further demonstrating the pseudo‐capacitive behavior of the electrode in the electrochemical reaction.^[^ ^15^ ^]^

As CuCo‐LDH@Cu/NPCW was applied with different current densities (2, 5, 10, 20, 30, and 50 mA cm^−2^, respectively), the area specific capacitances of the electrode were 26.24, 22.67, 19.87, 14.87, 11.07, and 8.33 F cm^−2^, respectively, and the mass specific capacitances were 749.71, 647.71, 567.71, 424.86, 316.29, and 238 F g^−1^ (Figure 4e), which were superior to most carbon‐based/CuCo‐based electrodes (Figure S15 and Table S4, Supporting Information). Significantly, the capacitance retention of CuCo‐LDH@Cu/NPCW was as high as 75.2% even when the current density was increased to 10 mA cm^−2^, with an excellent multiplicative performance. This was attributed to the self‐supported electrode structure and uniform pore structure facilitating electrolyte transport. The electrochemical impedance spectra (EIS) of the electrodes are shown in Figure 4f. All electrodes showed small equivalent series resistance Rs (<1 Ω). For example, CuCo‐LDH@Cu/NPCW had the smallest Rs of 0.70 Ω. The semicircle diameter of the EIS curve of CuCo‐LDH@Cu/NPCW in the high‐frequency region was also relatively small, indicating it had a low charge‐transfer resistance (Rct) (Table S5, Supporting Information). In addition, CuCo‐LDH@Cu/NPCW had the largest straight‐line slope in the low‐frequency region associated with the Warburg impedance (Rw), indicating CuCo‐LDH@Cu/NPCW had the fastest ion diffusion rate and the lowest ion diffusion resistance in the redox reaction.^[^ ^49^ ^]^ The results demonstrated that the formed “carbon‐metal‐LDH” tertiary conductive network provided an excellent interlayer structure and conductivity, which accelerated the ion diffusion rate and charge transfer efficiency.

The reaction kinetic behavior of CuCo‐LDH@Cu/NPCW with a hierarchical porous structure was verified by linearly fitting the anodic and cathodic peak currents of the CV curves (Figure S16, Supporting Information) at different scan rates by Equations 7 and 8.^[^ ^49^ ^]^

(7) $$i=av^{b}$$

(8) $$log\left(i\right)=log\left(a\right)+blog\left(v\right)\;$$

where a and b are computable parameters, v is the scan rate, and i is the peak current.

Typically, capacitance and diffusion synergistically control the energy storage process at the electrodes. When b = 0.5, the reaction process was mainly determined by diffusion control. Whereas as b approaches 1, it was dominated by capacitance‐control. The b‐values of the anodic and cathodic peaks of CuCo‐LDH@Cu/NPCW were calculated as 0.62 and 0.57, respectively (Figure 4g), indicating the dual control process of capacitance and diffusion in CuCo‐LDH@Cu/NPCW. Furthermore, the contribution ratio of diffusion to the capacitance control was calculated by Equations 9 and 10:^[^ ^52^ ^]^

(9) $$i\left(v\right)=k_{1}v+k_{2}\;v^{1/2}$$

(10) $$i\left(v\right)/v^{1/2}=k_{1}v^{1/2}+k_{2}\;$$

where k_1_v denotes the capacitance‐control contribution and k_2_v^1/2^ denotes the diffusion‐control contribution.

As shown in Figures 4h and S17 (Supporting Information), the ratio of the red region to the green region can be clearly expressed as the contribution. The capacitance‐control contribution was relatively small (32.00%) at a low scan rate (5 mV s^−1^). Figure 4i shows the capacitance‐control contribution at different scan rates, which increased from 22.30% to 64.00% as the scan rate increased from 1 to 40 mV⋅s^−1^. The result was attributed to the heterogeneous structure of “conducting skeleton‐active nanosheets” formed by Cu/NPCW and CuCo‐LDH nanosheets, which synergistically enhanced the double‐layer capacitance and pseudocapacitance. Cu/NPCW acted as a conductive backbone to provide double‐layer capacitance and fast charge transport, while CuCo‐LDH nanosheets acted as pseudocapacitive materials, which synergistically enhanced the capacitance.

Figure S18 (Supporting Information) illustrates that CuCo‐LDH‐5@Cu/NPCW had the largest integrated area of the CV curve, the longest discharge time, and the smallest charge transfer resistance. The recycling tests of CuCo‐LDH@Cu/NPCW were conducted at 30 mA cm^−2^ (Figure S19, Supporting Information). The results revealed that the capacitance retention of the electrode reached 96.70% after 10 000 recycles, indicating its excellent reversibility and stability. In addition, the structural and chemical states of CuCo‐LDH@Cu/ NPCW after long‐term cycling stability tests were investigated. No obvious morphological changes of CuCo‐LDH@Cu/NPCW were observed in Figure S20 (Supporting Information). A weak increase in the characteristic peaks of CuO and Co_3_O_4_ was observed in Figure S21 (Supporting Information), which was due to the partial oxidation of the catalyst during the cyclic stability test. In addition, as shown in the XPS spectra (Figure S22, Supporting Information), the CuCo‐LDH@Cu/NPCW maintained the same chemical elements, lattice planes, and chemical bonding states after the stability test as those before the cycling test, which indicated that the CuCo‐LDH@Cu/NPCW had excellent cycling stability.

**Figure 5:**
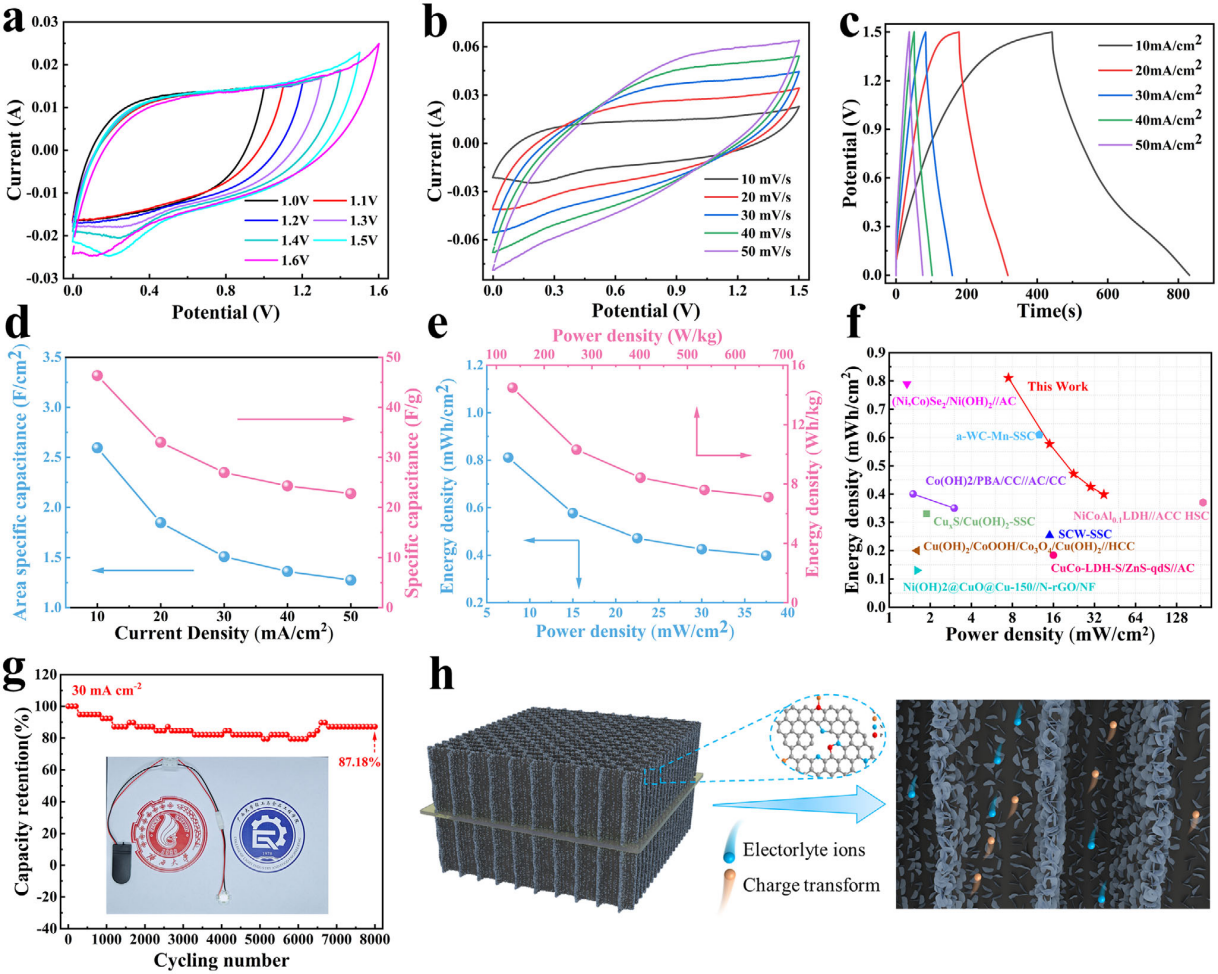
Capacitive performance of SSC: a) CV curves of the SSC devices at different potential ranges; b) CV curves at 10 to 50 mV s^−1^; c) GCD curves at 10 to 50 mA cm^−2^; d) Multiplicative performance based on different current densities; e) Power and energy density plot of the SSC; and f) The Ragone plot of the SSC for the comparison with the reported supercapacitor devices;^[^ ^1^, ^12^, ^53^, ^54^, ^55^, ^56^, ^57^, ^58^, ^59^ ^]^ g) Cycling stability of the SSC for 8000 cycles at 30 mA cm^−2^; h) Schematic representation of ion and electron transfer mechanisms within the SSC device.

In order to evaluate the practical applicability of electrodes in supercapacitors, the symmetric supercapacitor (SSC) device was assembled by CuCo‐LDH@Cu/NPCW, 2 M KOH as electrolyte, and a filter paper diaphragm. The SSC device had a voltage window of up to 1.5 V. At a voltage window of 1.5 V, the CV curve did not show significant polarization (**Figure** **5**a).^[^ ^51^ ^]^ The CV curve maintained a good rectangular shape as the scanning speed increased (Figure 5b), demonstrating reversible charging and discharging capability and excellent cycling reversibility. Meanwhile, the GCD curves of the SSC devices showed good symmetry at different current densities (Figure 5c). The specific capacitance of the SSC device was calculated as 2.60 F cm^−2^ (13 F cm^−3^ and 46.40 F g^−1^) at 10 mA cm^−2^. Even though the current density was increased to 50 mA cm^−2^, the SSC still achieved a specific capacitance of 1.29 F cm^−2^ (6.50 F cm^−3^ and 23.10 F g^−1^) with a capacitance retention of up to 50%, demonstrating its outstanding rate performance. It is noteworthy that the energy density of SSC reached 0.80 mWh cm^−2^ (4.10 mWh cm^−3^ and 14.50 Wh kg^−1^) at a power density of 7.50 mW cm^−2^ (37.50 mW cm^−3^ and 133.90 W kg^−1^). Notably, the assembled devices outperformed the reported carbon‐based/CuCo‐based supercapacitors (Figure 5f). The recycle tests were performed at a current density of 30 mA cm^−2^, the capacitance retention of SSC can retain 87.20% after 8000 recycles (Figure 5g). In addition, two series‐connected SSC devices can power an LED (1.0 W) for 20 min. The results demonstrated the great potential of CuCo‐LDH@Cu/NPCW in practical applications. Figure 5h illustrates the potential mechanism for the excellent capacitance performance of the SSC device prepared with CuCo‐LDH@Cu/NPCW.

### 2.3 HER Property

**Figure 6:**
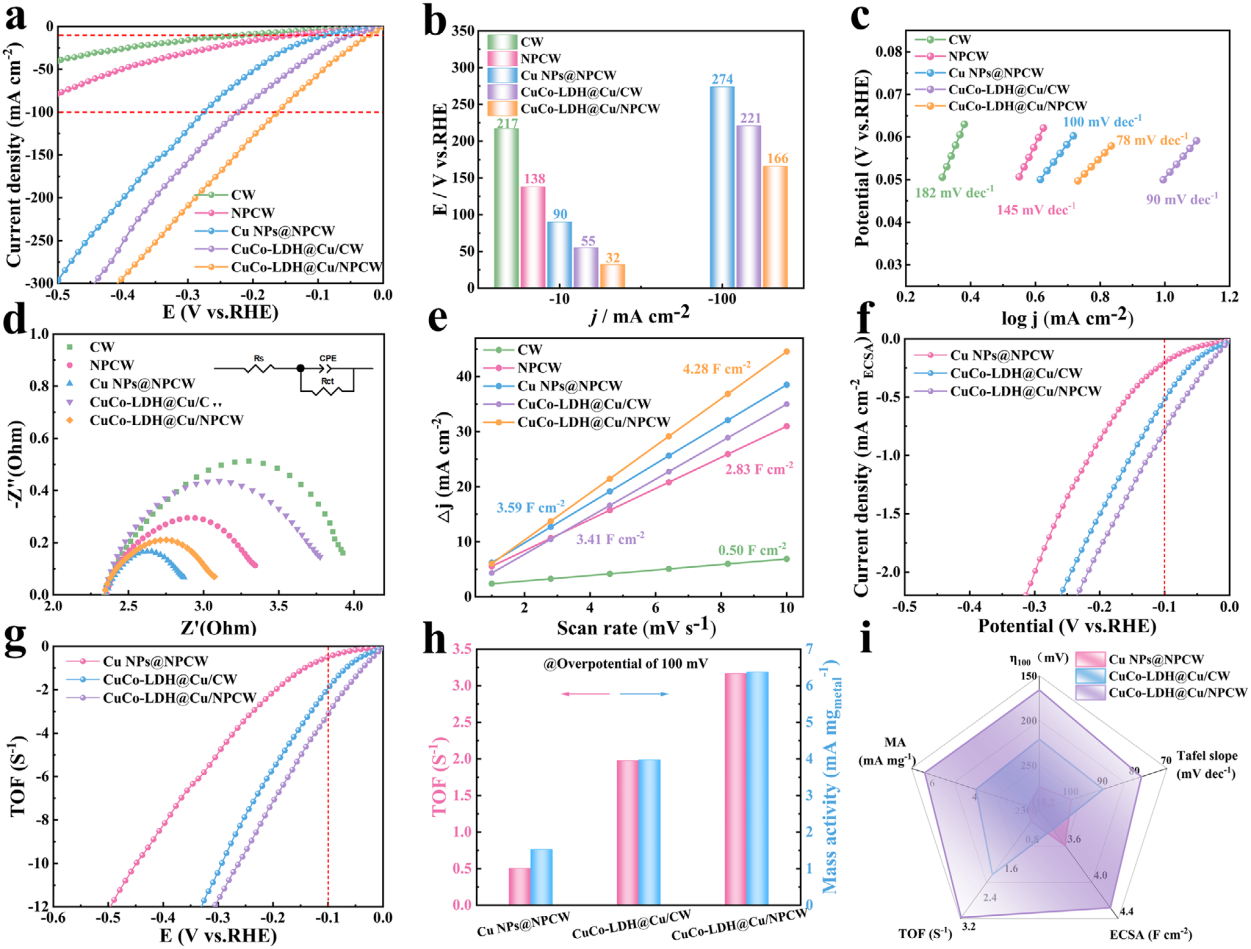
a) The HER polarization curves of electrocatalysts; b) Comparison of the overpotentials required at 10 and 100 mA cm^−2^ of different catalysts; c) Tafel plots; d) EIS spectra; e) Cdl values; f) LSV curves of the normalized electrochemical active area; g) TOF curves; h) The TOF and mass activity at a 100 mV overpotential; i) Integrated Performance Radar Chart.

The alkaline HER activity of the catalysts in a three‐electrode system was performed with 1.0 M KOH solution as the electrolyte. As shown in **Figure** **6**a,b, the CuCo‐LDH@Cu/NPCW electrode exhibited the most excellent HER overpotential, which was only 32 and 166 mV at 10 and 100 mA cm^−2^, respectively. Impressively, the NPCW had an overpotential of 138 mV at 10 mA cm^−2^, which was significantly better than that of CW (217 mV). CuCo‐LDH@Cu/CW showed a significantly improved overpotential of 55 mV at 10 mA cm^−2^. The results confirmed that the introduction of CuCo‐LDH nanosheets and the N/P co‐doping significantly enhanced the catalytic performance. This was attributed to the synergistic effect of Cu/NPCW and CuCo‐LDH nanosheets modulating the electronic structure of the catalysts, optimizing the H* adsorption free energy, and improving their intrinsic catalytic activity. In addition, Figure 6c shows that the Tafel slope of CuCo‐LDH@Cu/NPCW was only 78 mV dec^−1^, demonstrating that the HER‐catalyzed process at the electrode was determined by the Volmer‐Heyrovsky mechanism.^[^ ^60^ ^]^ Notably, the Tafel slope values of CW, NPCW, Cu NPs@NPCW, and CuCo‐LDH@ Cu/CW were significantly higher than those of CuCo‐LDH@Cu/NPCW, confirming that the introduction of CuCo‐LDH nanosheets and the N/P co‐doping elevated the reaction kinetic rate.

As shown in Figure 6d, CuCo‐LDH@Cu/NPCW had the smallest charge transfer resistance (Rct) compared to CW, NPCW, and CuCo‐LDH@Cu/CW, indicating CuCo‐LDH@Cu/NPCW had the fastest charge transfer rate and highest catalyst intrinsic activity,^[^ ^24^ ^]^ which was in good agreement with the Tafel slope. The C_dl_ values of the electrodes were calculated by testing the cyclic voltammetry curves at different scan rates (Figure S23, Supporting Information). As shown in Figure 6e, the Cdl value of CuCo‐LDH@Cu/NPCW was 4.28 F cm^−2^, which was much higher than that of CW (0.50 F cm^−2^), NPCW (2.83 F cm^−2^), Cu NPs@NPCW (3.59 F cm^−2^), and CuCo‐LDH@Cu/CW (3.41 F cm^−2^), revealing CuCo‐LDH@Cu/NPCW had more electrochemically active centers and stronger catalytic activity. In addition, the HER electrocatalytic performance of CuCo‐LDH‐5@Cu/NPCW was significantly better than those of CuCo‐LDH‐1@Cu/NPCW, CuCo‐LDH‐3@Cu/NPCW, CuCo‐LDH‐7@Cu/NPCW, and CuCo‐LDH‐10@Cu/NPCW due to the formation of a uniform and effective hierarchical heterostructure by CuCo‐LDH nanosheets with Cu/NPCW, as well as the CuCo‐LDH nanosheets achieved the desired coverage (Figure S24, Supporting Information).

Figure 6f shows the ECSA‐normalized LSV curves to further illustrate the intrinsic activity of the catalysts. At the same potential, the normalized current density of CuCo‐LDH@Cu/ NPCW was the highest, indicating its highest catalytic efficiency (Figure S25, Supporting Information).^[^ ^77^ ^]^ As shown in Figure 6g,h, the TOF (3.20 s^−1^) and mass activity (6.40 mA mg^−1^) of CuCo‐LDH@Cu/NPCW were much higher than those of Cu NPs@NPCW (0.50 s^−1^, 1.50 mA mg^−1^) and CuCo‐LDH@Cu/CW (2.00 s^−1^, 4.00 mA mg^−1^) at the overpotential of 100 mV, implying CuCo‐LDH@Cu/NPCW had high intrinsic activity against HER.^[^ ^41^ ^]^ Radar plots showed the comprehensive comparison of catalyst performance in terms of overpotential, turnover frequency (TOF), electrochemically active surface area (ECSA), Tafel slope, and mass activity, indicating that CuCo‐LDH@Cu/NPCW outperformed the other catalysts (Figure 6i).

**Figure 7:**
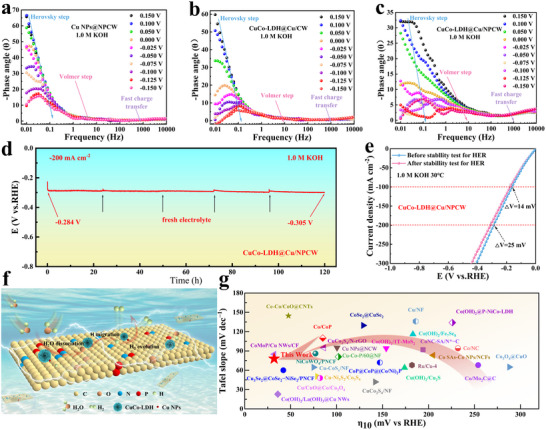
a–c) In situ EIS tests of Cu NPs@NPCW, CuCo‐LDH@Cu/CW and CuCo‐LDH@Cu/NPCW for HER; d) Chronopotentiometry (CP) curves of CuCo‐LDH@Cu/NPCW at 200 mA cm^−2^; e) LSV curve of CuCo‐LDH@Cu/NPCW before and after CP stability test; f) Schematic diagram of electrocatalytic hydrogen reaction of CuCo‐LDH@Cu/NPCW; g) Comparison of the overpotential required to generate 10 mA cm^−2^ current density together with the Tafel slope of CuCo‐LDH@Cu/NPCW and the reported catalysts.^[^ ^15^, ^61^, ^62^, ^63^, ^64^, ^65^, ^66^, ^67^, ^68^, ^69^, ^70^, ^71^, ^72^, ^73^, ^74^, ^75^, ^76^ ^]^

The Bode plots of CuCo‐LDH@Cu/NPCW showed a significant reduction of the frequency peaks and shift to higher frequencies as the applied potential decreased (**Figure** **7**a–c). This was attributed to the decrease in the charge transfer resistance at the electrolyte‐catalyst interface and the increase in the interfacial reaction rate. In addition, electron transfer occurred only at the Volmer step (in the mid‐frequency region) and the Heyrovsky step (in the low‐frequency region), and the Tafel step was difficult to show electron transfer by EIS.^[^ ^41^, ^77^ ^]^ Phase angles were observed in the low, medium, and high‐frequency regions for Cu NPs@NPCW, CuCo‐LDH@Cu/CW, and CuCo‐LDH@Cu/NPCW, indicating that HER followed the Volmer‐Heyrovsky mechanism. Interestingly, the phase angle of CuCo‐LDH@Cu/NPCW decreased significantly with the increasing voltage in the low and medium frequency regions, and finally approached zero. This result indicated that the reaction intermediate processes, such as H*/OH* absorption, were optimized and the reaction kinetics were accelerated through the construction of the heterostructure of “conducting skeleton‐active nanosheets” (Figure 7c). The stability tests revealed that the potential of CuCo‐LDH@Cu/NPCW did not significantly change at a current density of −200 mA cm^−2^ for up to 120 h (Figure 7d). After the stability test, the polarization curves were almost overlapped (Figure 7e), verifying their excellent stability. Moreover, the crystal structure and morphology of CuCo‐LDH@Cu/ NPCW were analyzed through XRD and SEM after the HER catalytic performance test. As shown in Figures S26,S27, and S28 (Supporting Information), the crystal structure and morphology of CuCo‐LDH@Cu/ NPCW were well preserved, indicating its outstanding electrochemical stability. As a result, the HER performance of CuCo‐LDH@Cu/NPCW was significantly better than most carbon‐based/CuCo‐based electrodes (Figure 7g; Table S6, Supporting Information).

As a result, a synergistic mechanism was proposed in Figure 7f. NPCW provides the basic conducting skeleton for CuCo‐LDH@Cu/ NPCW, and the N/P co‐doping active surface provides abundant sites and suppresses the agglomeration of the catalyst. The Cu NPs act as the nucleation sites for in situ growing of LDH, thereby developing a stable “carbon‐metal‐LDH” tertiary conducting network. During the electrocatalytic reaction, water molecules are adsorbed and dissociated at the Co sites (Volmer step) to produce H species, which then are transferred from the Co sites to the Cu sites, where the adsorbed H* is converted into H_2_ (Heyrovsky step). Therefore, the synergistic effect on HER is endowed.^[^ ^78^, ^79^ ^]^

## 3 Conclusion

CuCo‐LDH@Cu/NPCW with a hierarchical porous structure and an array of ultrathin nanosheets was successfully developed, which possessed excellent supercapacitive and HER performance. The specific capacitance was as high as 26.24 F cm^−2^ (749.71 F g^−1^) at 2 mA cm^−2^, and the capacitance retention was up to 96.7% after 10000 cycle tests. The assembled symmetric supercapacitor (SSC) achieved a high energy density of 0.80 mWh cm^−2^ at 7.50 mW cm^−2^. In addition, CuCo‐LDH@Cu/NPCW exhibited outstanding catalytic activity for HER. The overpotentials of CuCo‐LDH@Cu/NPCW were as low as 32 mV at 10 mA cm^−2^ and 166 mV at 100 mA cm^−2^. The electrode demonstrated remarkable stability with no significant changes in overpotential in a 120 h continuous hydrogen production test. Furthermore, the synergistic mechanism of Cu/NPCW and CuCo‐LDH nanosheets was proposed. The findings pave a new pathway for the synthesis and performance of biomass carbon‐derived composites for dual applications as supercapacitors and HER.

## Conflict of Interest

The authors declare no conflict of interest.

## Supporting information



Supporting Information

## Data Availability

The data that support the findings of this study are available from the corresponding author upon reasonable request.
