# Supplementary material for: CuCo‐Layered Double Hydroxide Nanosheets Grown on Hierarchical Carbonized Wood as Bifunctional Electrode for Supercapacitor and Hydrogen Evolution Reaction
Source: Adv Sci (Weinh). 2025 Sep 8;12(45):e08630. doi: 10.1002/advs.202508630 (PMC12677625; doi:10.1002/advs.202508630)
Supplement: Supplementary file 1 — Supporting Information [file ADVS-12-e08630-s001.docx]

**Supporting Information**

**CuCo-LDH Nanosheets Grown on Hierarchical Carbonized Wood as Bifunctional Electrode for Supercapacitor and Hydrogen Evolution Reaction**

Hewei Hou ^1^, Guiqing Lei ^1^, Huashuang Huo ^1^, Yuanyuan Yu ^1^, Zhenzhen Tang ^1^, Chengrong Qin ^1^, and Douyong Min ^1, *^

^1^ Guangxi Key Laboratory of Clean Pulp & Papermaking and Pollution Control, School of Light Industry and Food Engineering, Guangxi University, Nanning 530004, China

The corresponding author’s email: mindouyong@gxu.edu.cn

**1. Experimental Section**

**1.1 Materials**

The softwood (pine wood) was purchased from Linyi Beimu Wood Products Co., Ltd (Linyi, China). High-purity nitrogen (N_2_, 99.999%) and high-purity argon (Ar, 99.999%) were provided by Guangxi Ruida Chemical Technology Co., Ltd. (China). Concentrated hydrochloric acid (HCl, 37 wt%) was purchased from Nanning Lantian Experimental Equipment Co., Ltd. (China). Ammonium dihydrogen phosphate (NH_4_H_2_PO_4,_ 99.5 wt%), anhydrous ethanol (Ethanol 98 wt%), copper chloride dihydrate (CuCl_2_ 2H_2_O, 99 wt%), urea (CO(NH_2_)_2_, 95 wt%), potassium hydroxide (KOH, 85 wt%) and cobalt nitrate hexahydrate (Co(NO_3_)_2_·6H_2_O, 99 wt%) were purchased from Nanning Rongyi Experimental Equipment Co., Ltd. (China). The reagents were used without further purification.

**1.2 Preparation of NPCW**

The pine wood was cut perpendicular to its longitudinal direction into slices (20 mm × 20 mm × 2.5 mm, radial × tangential × longitudinal) using a bandsaw machine and then pre-oxidized in an air atmosphere at 220 ℃ for 4 h (1 ℃/min) in a blast-drying oven. The pre-oxidized wood slices were mixed with NH_4_H_2_PO_4_ at a mass ratio of 1:1.25 and then impregnated with 10 mL of deionized water and evaporated at 60 °C in a vacuum oven. The dried wood slices were transferred to a tube furnace and carbonized at 1000 °C for 3 h (5 ℃/min, argon flow rate of 100 sccm) under a nitrogen atmosphere to obtain N/P co-doped carbonized wood, denoted as NPCW.

**1.3 Preparation of Cu NPs@NPCW**

The nitrogen-phosphorus co-doped carbonized wood was polished with 2000-mesh sandpaper and then cut into 15 mm × 15 mm × 1 mm electrodes, which were ultrasonically washed by 0.5 M hydrochloric acid for 15 min, ethanol for 60 min, and water for 30 min, and then dried under vacuum at 60 ℃ for 10 h. The polished NPCW was impregnated with a 1.0 M CuCl_2_ solution in a vacuum for 12 h. Afterwards, it was transferred to a tube furnace and annealed at 1000 °C for 3 h (5 ℃/min, argon flow rate of 100 sccm) under argon atmosphere to grow copper nanoparticles on NPCW (named as Cu NPs@NPCW).

**1.4 Preparation of CuCo-LDH@Cu/NPCW**

Firstly, 5 mmol Co(NO_3_)_2_·6H_2_O and 10 mmol urea were dissolved in 30 mL anhydrous ethanol and stirred for 30 min. Then, the as-prepared solution and Cu NPs@NPCW were transferred to a 50 ml Teflon high-temperature reactor, and Cu NPs@NPCW were vertically immersed in the solution. Subsequently, it was transferred to an oven and heated at 90 °C for 10 h to grow copper-cobalt double hydroxide on Cu NPs@NPCW, which was named as CuCo-LDH@Cu/NPCW. The electrodes prepared with different Co loadings by different amounts of Co(NO_3_)_2_·6H_2_O (1, 3, 7, and 10 mmol) under the same conditions, which were denoted as CuCo-LDH-y@Cu/NPCW (y represents the amount of Co(NO_3_)_2_·6H_2_O).

**1.5 Material characterization**

The Brunauer-Emmett-Teller (BET) surface analyzer (MICROMERITICS, ASAP 2460, U.S.A.) was used to calculate the surface area and pore size via nitrogen adsorption−desorption isotherms. Before the test, the samples (50 mg) were degassed under vacuum at 200 °C for 12 h, then cooled to room temperature and purged with nitrogen. The crystalline phases of the samples were obtained by an X-ray diffractometer (XRD, MINFLEX600, Japan) operated in the range of 5-100° using Cu Kα radiation (λ = 1.5418 Å) at a voltage of 40 kV and a current of 30 mA. Raman spectroscopy was recorded by a laser Raman spectrometer (Raman, Via Reflex, UK, laser wavelength 532 nm) using a 50× long working distance visible objective during Raman tests and calibrated with a silicon standard sample (520.7 cm^-1^). The hydrophilicity of the samples was determined by a Contact Angle Measuring Instrument (KRUSS, Germany). The surface valence states of the samples were tested by using an X-ray photoelectron spectrometer (XPS, Thermo Scientific K-Alpha, Al Kα radiation), and all XPS spectral results were corrected by the C 1s peak at 284.8 eV. The XPS samples were prepared as follows: First, the bulk samples were placed in an agate mortar and ground into powder. Subsequently, a small amount of the powdered sample was placed on a piece of aluminum foil, and the sample was pressed using a tablet press. Finally, the prepared sample was placed on a sample stage for analysis.

The metal atoms were detected using an inductively coupled plasma emission spectrometry analyzer (ICP-OES, Agilent 720ES). The morphology and elemental distribution of the samples were obtained by a scanning electron microscope (SEM, ZEISS sigma300, Germany) equipped with an energy dispersive spectrometer (EDS, Oxford Ultim Max40). Transmission electron microscopy (TEM) and high-resolution transmission electron microscopy (HRTEM) were performed under a transmission electron microscope (Thermo Fisher Talos F200X G2, USA) with an accelerating voltage of 200 kV. Initially, the bulk samples were dispersed in an ethanol solution after being fully ground into powder and sonicated for 10 min. Subsequently, droplets of the solution were deposited onto a molybdenum mesh coated with a carbon film using a pipette. After accelerated drying using an infrared lamp, the TEM samples were analyzed. TEM tests were performed at an operating voltage of 200 kV.

**1.6 Supercapacitor measurements**

A multichannel electrochemical workstation (INTERFACE 1000, U.S.A.) was used to perform tests such as cyclic voltammetry (CV), galvanostatic charging and discharging (GCD), and electrochemical impedance spectroscopy (EIS) in a typical three-electrode system in 2.0 M KOH electrolyte at room temperature. In the test process, Hg/HgO (1.0 M KOH solution) electrode, Pt electrode (20×20×1 mm^3^), and CuCo-LDH@Cu/NPCW electrode (10×10×1 mm^3^) were used as reference, counter, and working electrodes, respectively. The CV and GCD tests were measured in a 2.0 M KOH electrolyte at potentials ranging from -1 to 0 V with different scan rates and current densities. The EIS test was carried out at an open-circuit potential in the frequency range of 0.01 to 100 kHz with a voltage amplitude of 5 mV. The cyclic stability tests were performed on LANDCT2001A (China). The area specific capacitance (Cs, F cm^-2^), mass specific capacitance (Cm, F g^-1^), and volume specific capacitance (Cv, F cm^-3^) were calculated by Eqs. 1, 2, and 3, respectively.

$Cs=\left( I\times\Delta t \right)/\left( S\times\Delta V \right)$ (*Eq.* 1)

$Cm=\left( I\times\Delta t \right)/\left( m\times\Delta V \right)$ (*Eq.* 2)

$Cv={Cs}/d$ (*Eq.* 3)

Where, I (A), ∆V (V), ∆t (s), S (cm^2^), m (g), and d (cm) represent the discharge current, the value of the potential change during the discharge, the discharge time, the electrode area, the electrode mass and the electrode thickness, respectively.

The energy density (E) and power density (P) were calculated by Eqs. 4 and 5.

$E=\left( C\times{\Delta V}^{2} \right)/\left( 2\times3.6 \right)$ (*Eq.* 4)

$P={3600E}/{\Delta t}$ (*Eq.* 5)

Where ∆V (V) is the change value of the potential and ∆t (s) is the discharge time.

**1.7. Electrocatalytic measurements**

The HER measurements were performed at 30 °C in a 1.0 M KOH conventional three-electrode cell, as CuCo-LDH@Cu/NPCW and other reference samples were used as the working electrodes, while the graphite rod electrode and the Hg/HgO electrode were used as the counter electrode and the reference electrode, respectively. The potential of HER was calibrated against the reversible hydrogen electrode (RHE) using the following formula: E(RHE) = E(Hg/HgO) + 0.059 × pH + 0.098 V^[1]^. The electrodes were activated by cyclic voltammetry (CV) with 20 cycles at a scan rate of 50 mV s^-1^ until the surface electrochemical properties of the electrodes were stabilized. The HER performance was evaluated by linear voltammetry scanning (LSV) at a scan rate of 5 mV s^-1^. All polarization curves were 90% iR-corrected. Bilayer capacitance (C_dl_) and electrochemical specific surface area (ECSA) of the electrodes were obtained by CV in the non-Faraday zone. Notably, the C_dl_ of the catalyst was calculated from the following equation: C_dl_ = I/K, where K is the scan rate. The ECSA was calculated by Eq. 6, assuming a standard value of 60 mF cm^-2[2]^:

$ECSA={C_{dl}}/{60}$ (*Eq.* 6)

The turnover frequency (TOF) was calculated by Eq. 7.

$TOF={js}/{2Fn}$ (*Eq.* 7)

Where, j (mA cm^-2^) is the current density, S (cm^-2^) is the electrode area, F is the Faraday constant (96485C mol^-1^), and n refers to the number of moles of catalyst loaded on the NPCW.

The *in situ* EIS measurements of HER were carried out at different potentials in the frequency range of 10 kHz to 0.01 Hz. The stability test of the CuCo-LDH@Cu/NPCW electrode was performed using the chronopotential (CP) method.

**2. Supplementary Figures**


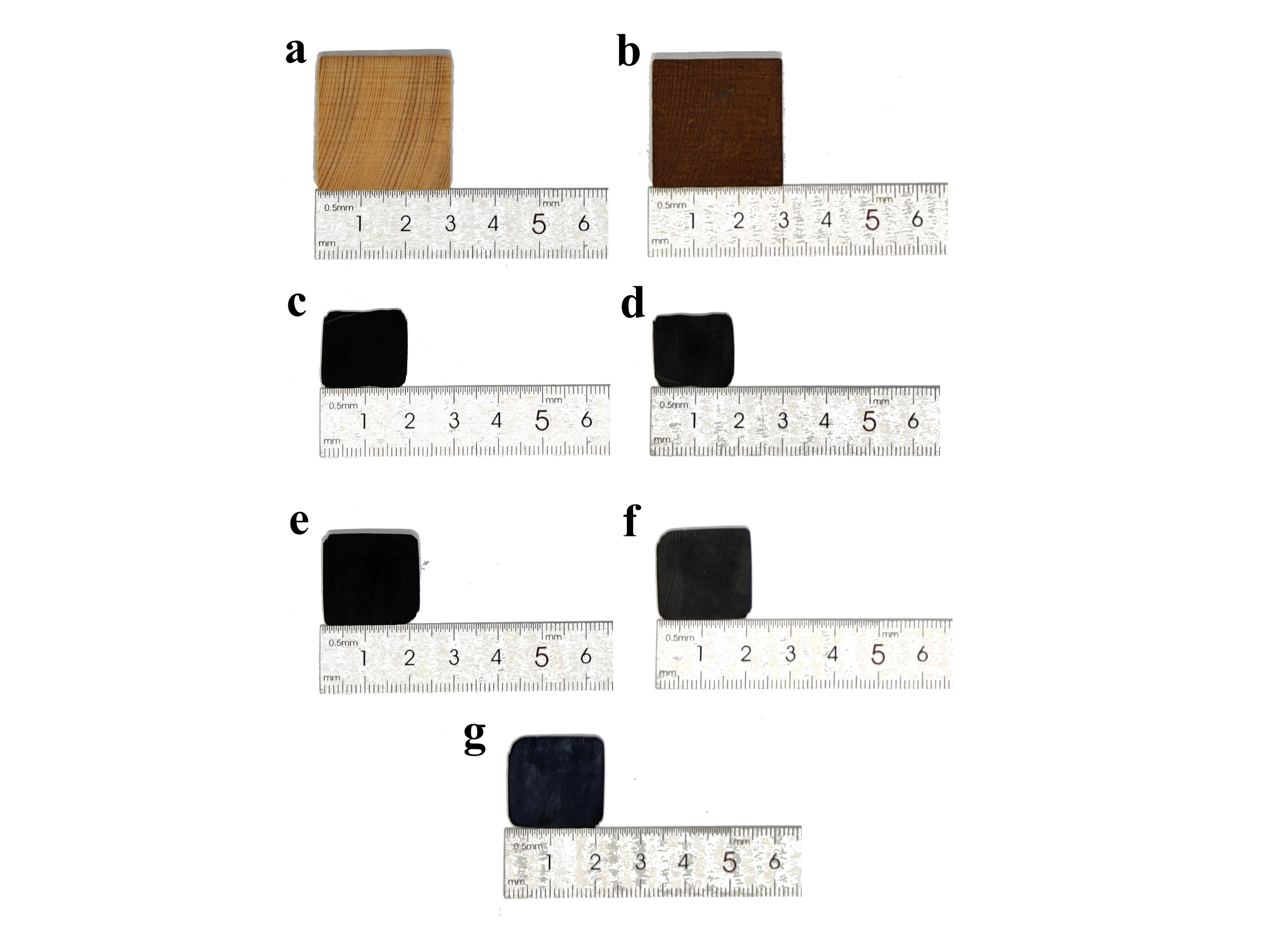


**Fig. S1** The photos of the (a) natural wood, (b) pre-oxidized wood, (c) CW, (d) CuCo-LDH@Cu/CW, (e) NPCW, (f) Cu NPs@NPCW, and (g) CuCo-LDH@Cu/NPCW samples.


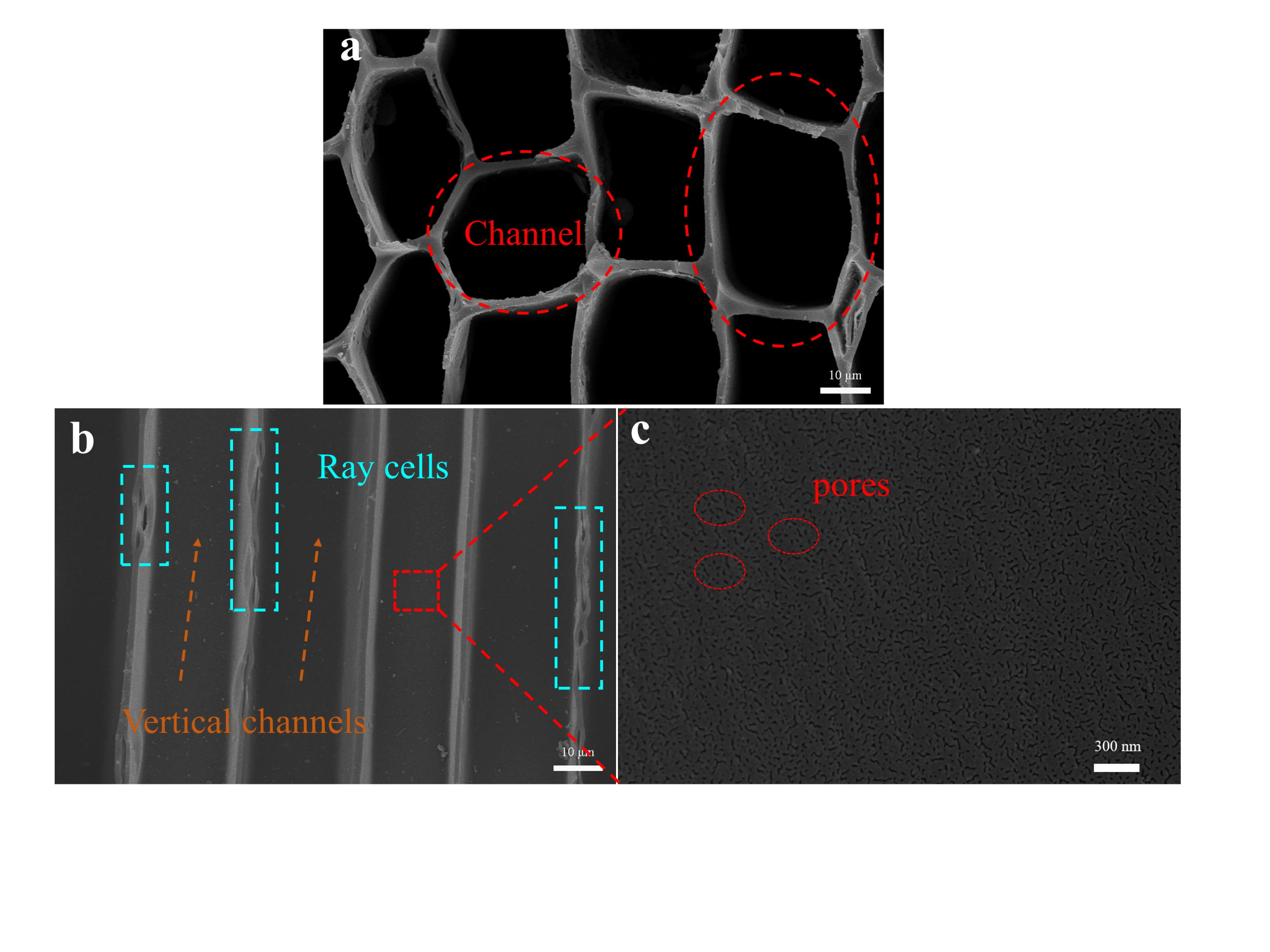


**Fig. S2** SEM images of NPCW: (a) cross section, (b) longitudinal section, (c) the corresponding magnified area.


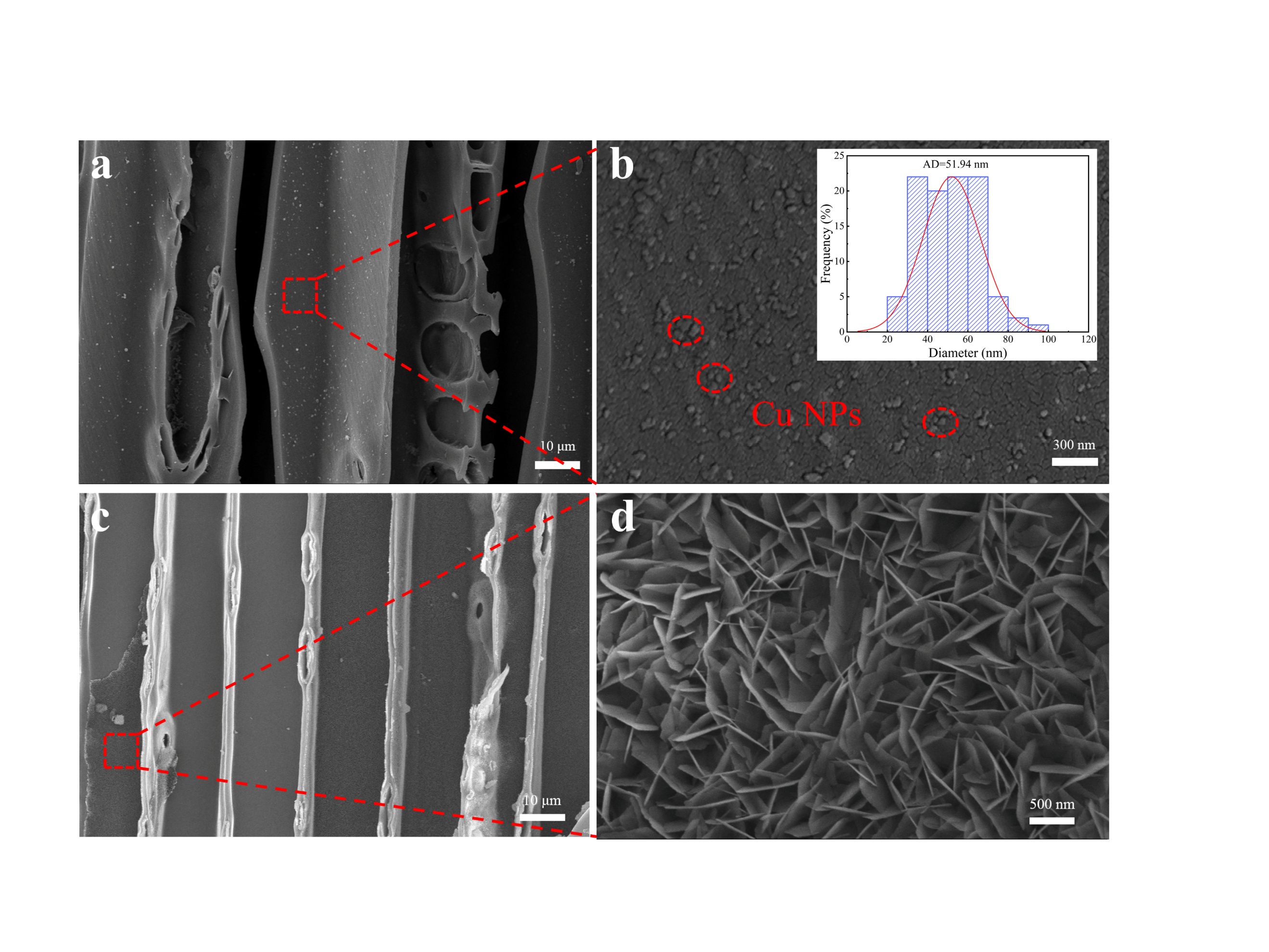


**Fig. S3** (a-b) SEM images of Cu NPs@NPCW; (c-d) SEM images of CuCo-LDH@Cu/CW.


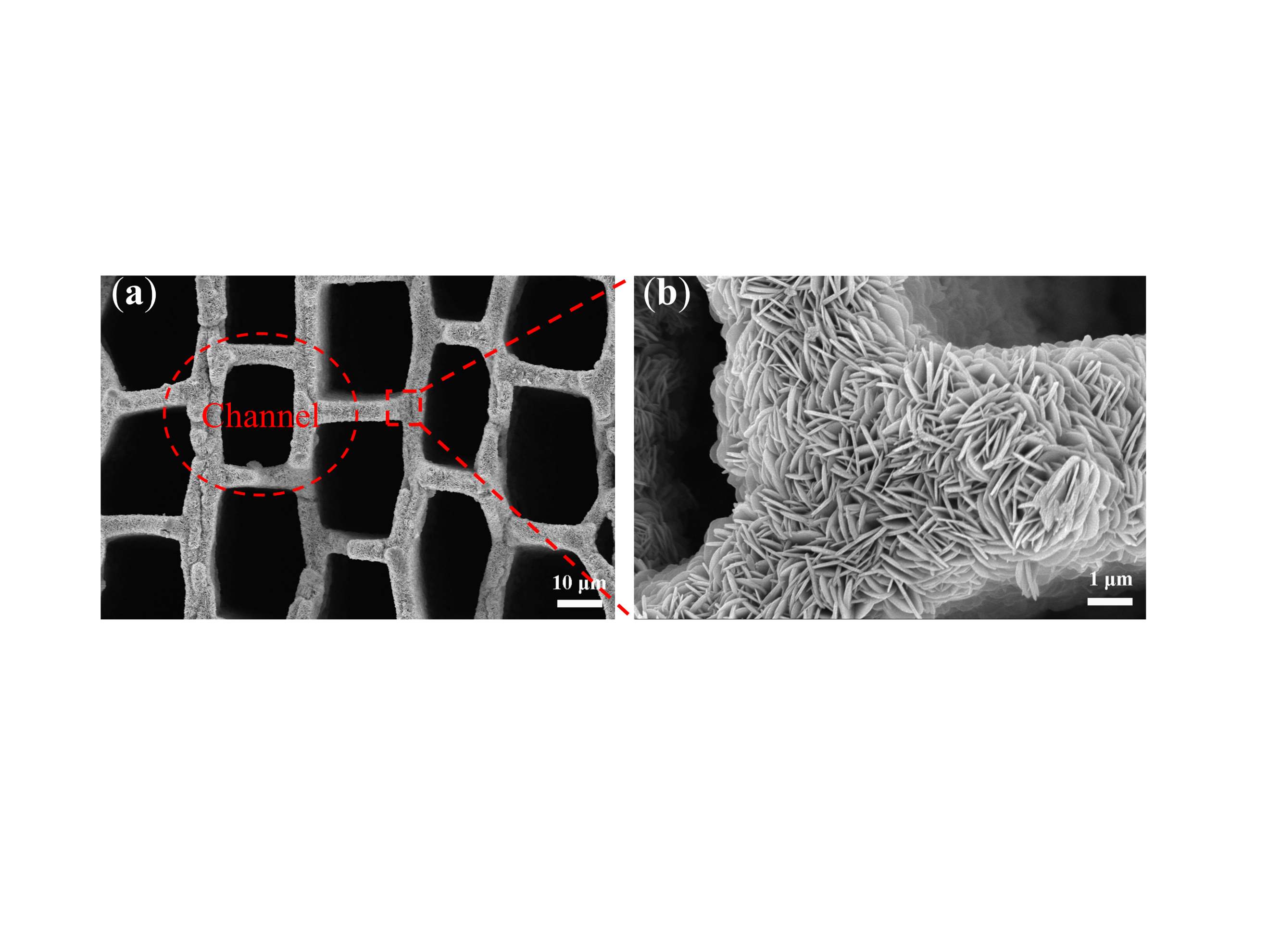


**Fig. S4** (a-b) SEM images of CuCo-LDH@Cu/NPCW, cross-section.


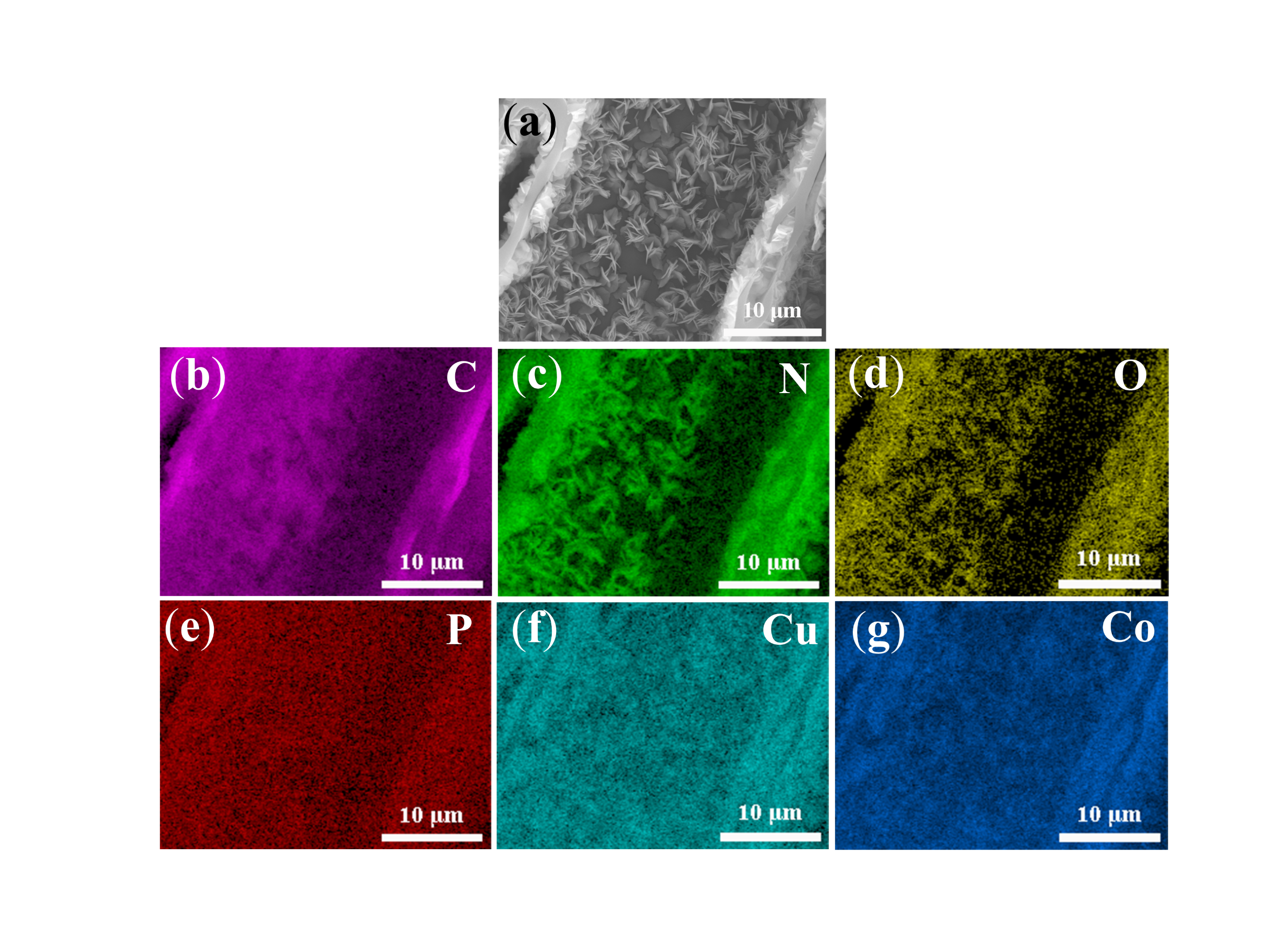


**Fig. S5** (a) SEM images of CuCo-LDH@Cu/NPCW; (b-g) Elemental mappings of C, N, O, P, Cu, and Co for CuCo-LDH@Cu/NPCW electrode.


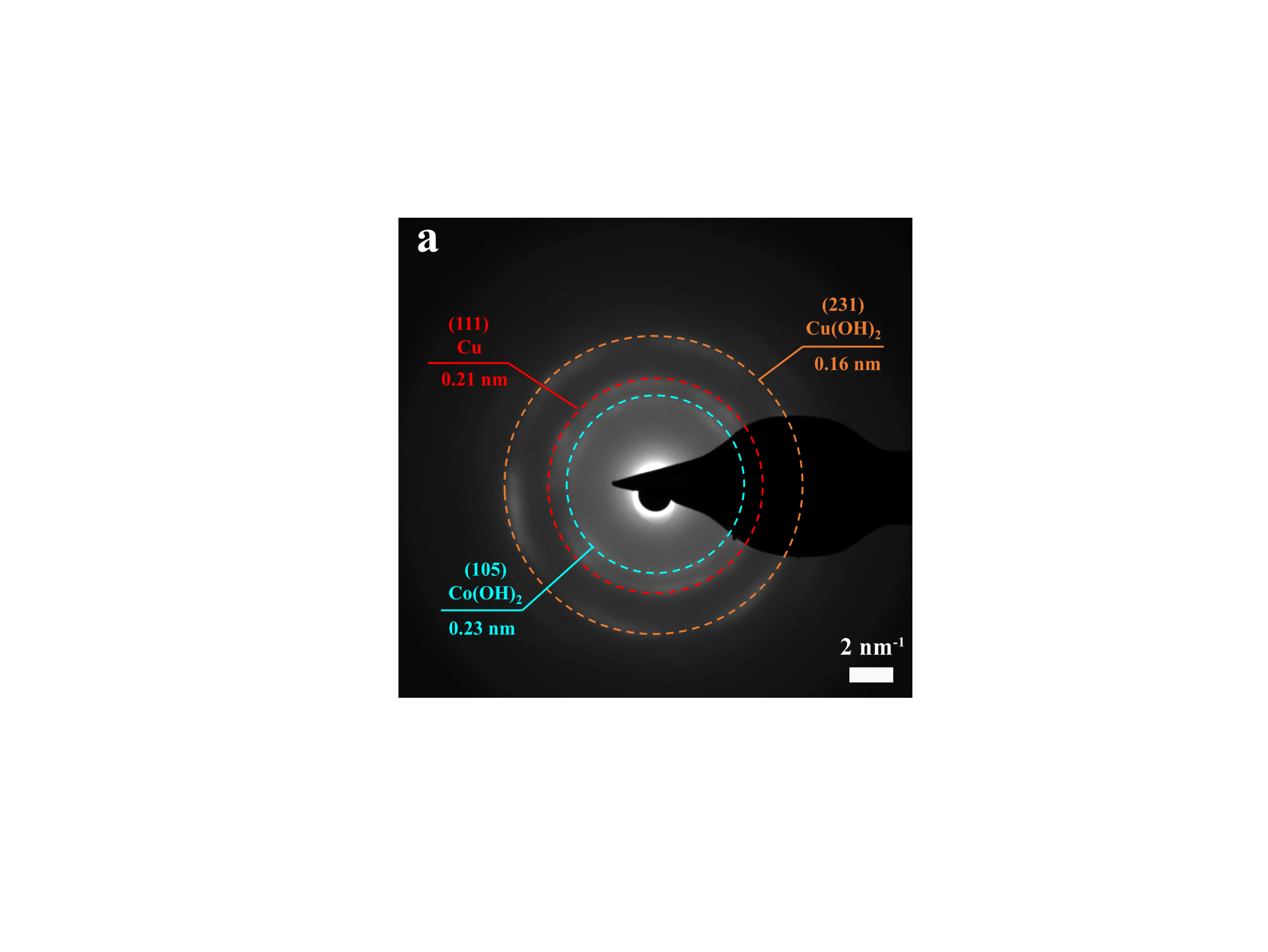


**Fig. S6** Selected area electron diffraction pattern of CuCo-LDH@Cu/NPCW.


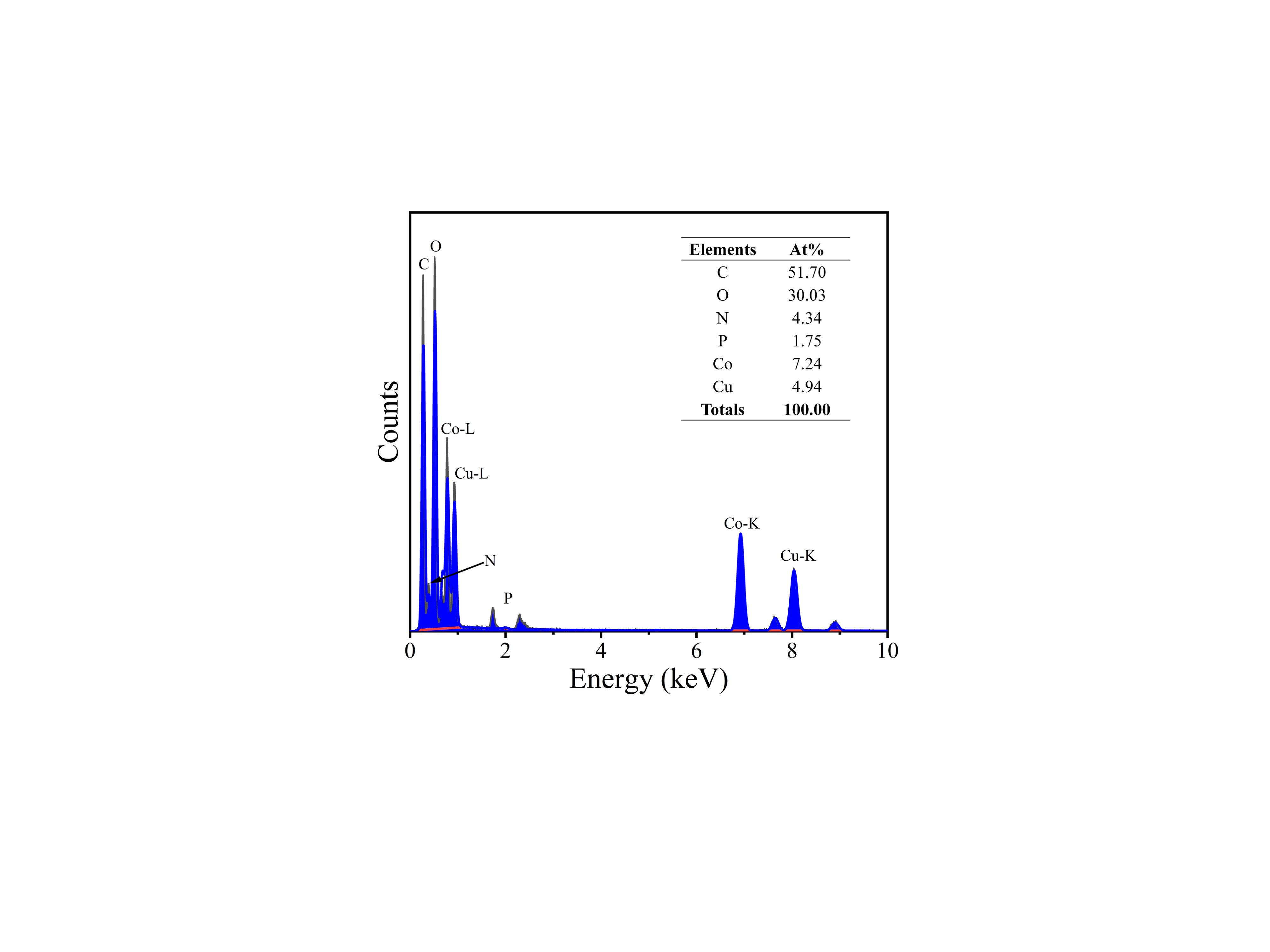


**Fig. S7** EDX spectrum of CuCo-LDH@Cu/NPCW.


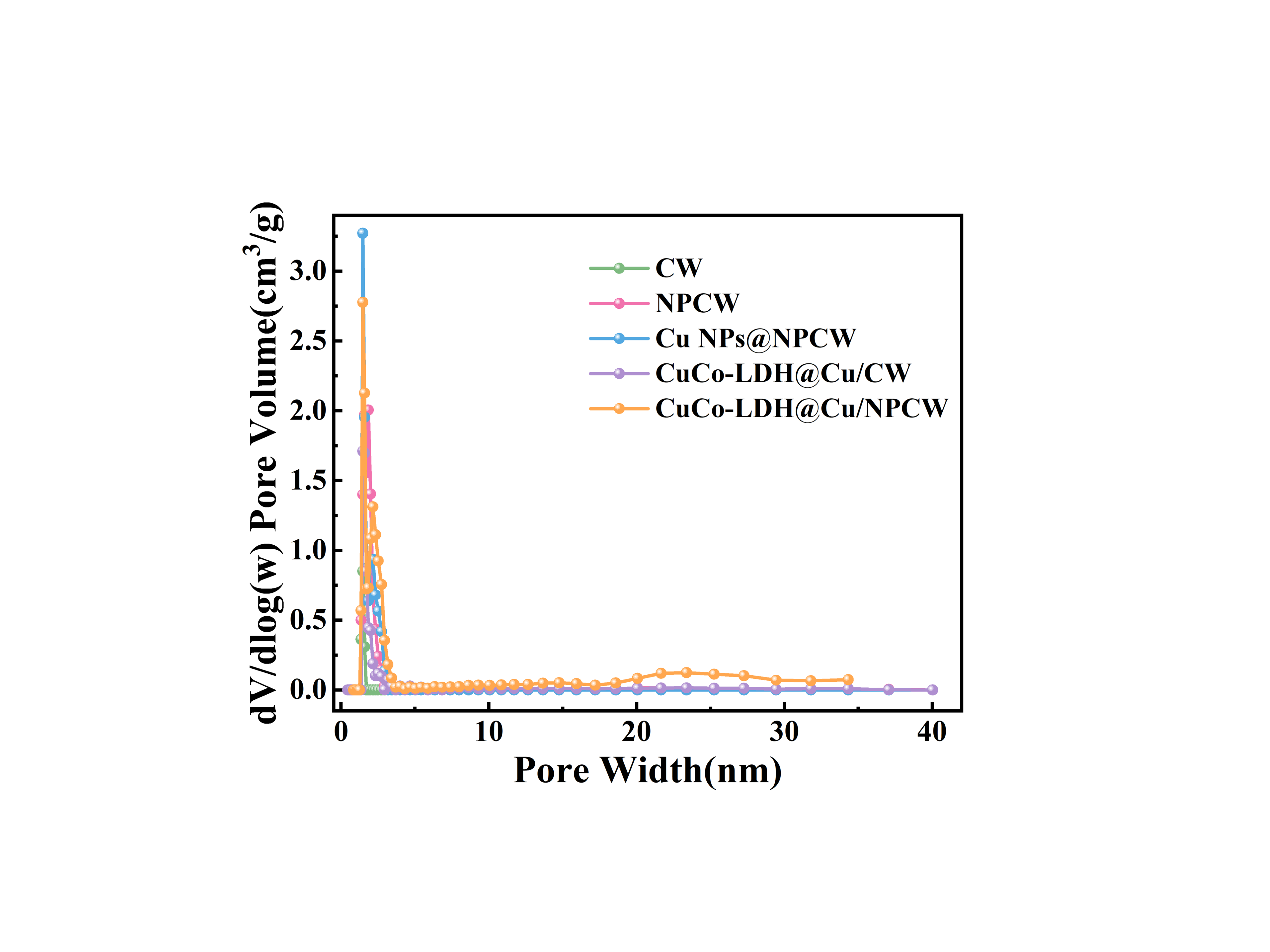


**Fig. S8** Pore size distribution of CW, NPCW, Cu NPs@NPCW, CuCo-LDH@Cu/CW, and CuCo-LDH@Cu/NPCW electrodes.


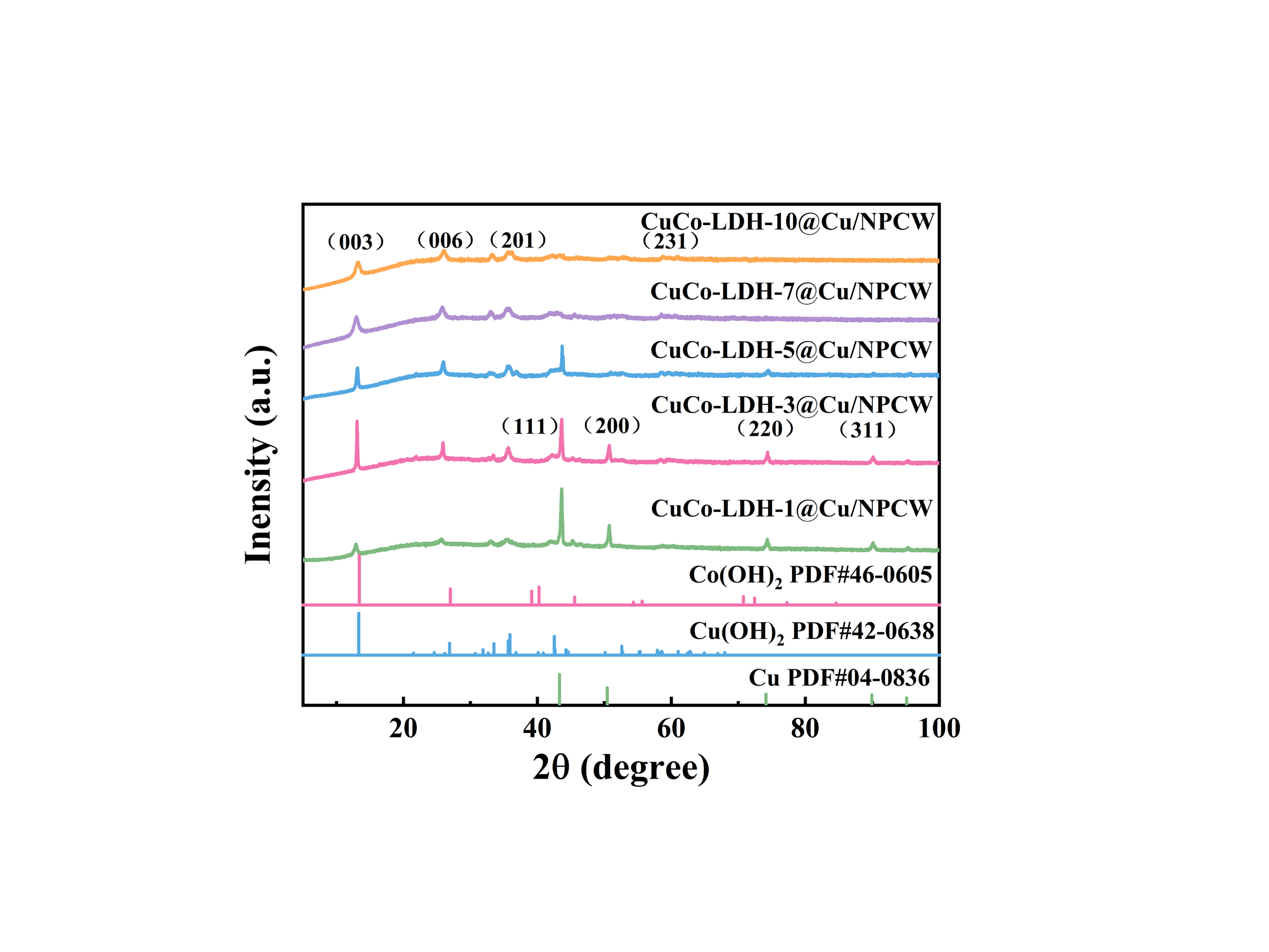


**Fig. S9** XRD spectra of CuCo-LDH-y@Cu/NPCW prepared with different Co^2+^.


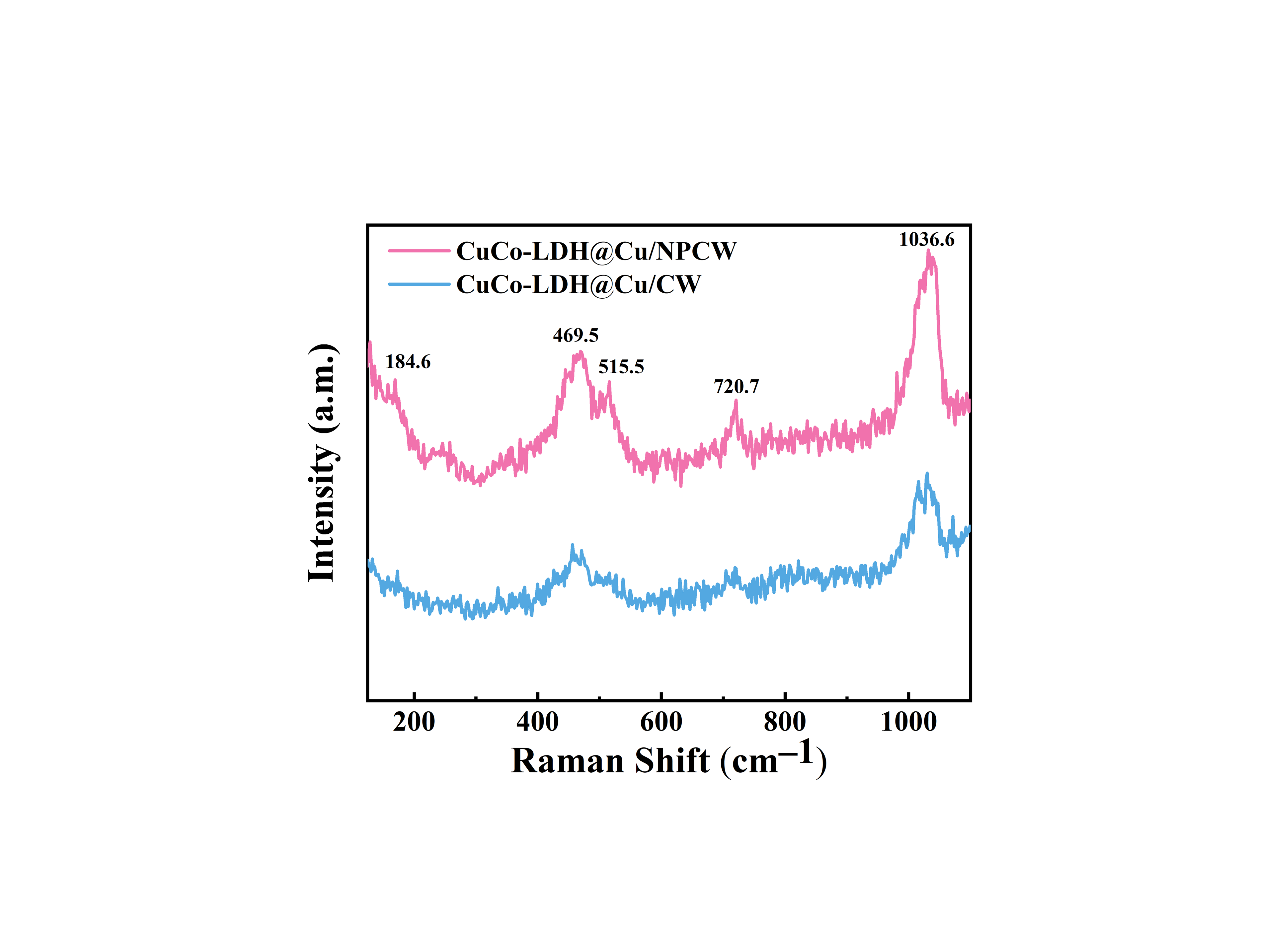


**Fig. S10** Raman patterns of CuCo-LDH@Cu/CW, and CuCo-LDH@Cu/NPCW electrodes.


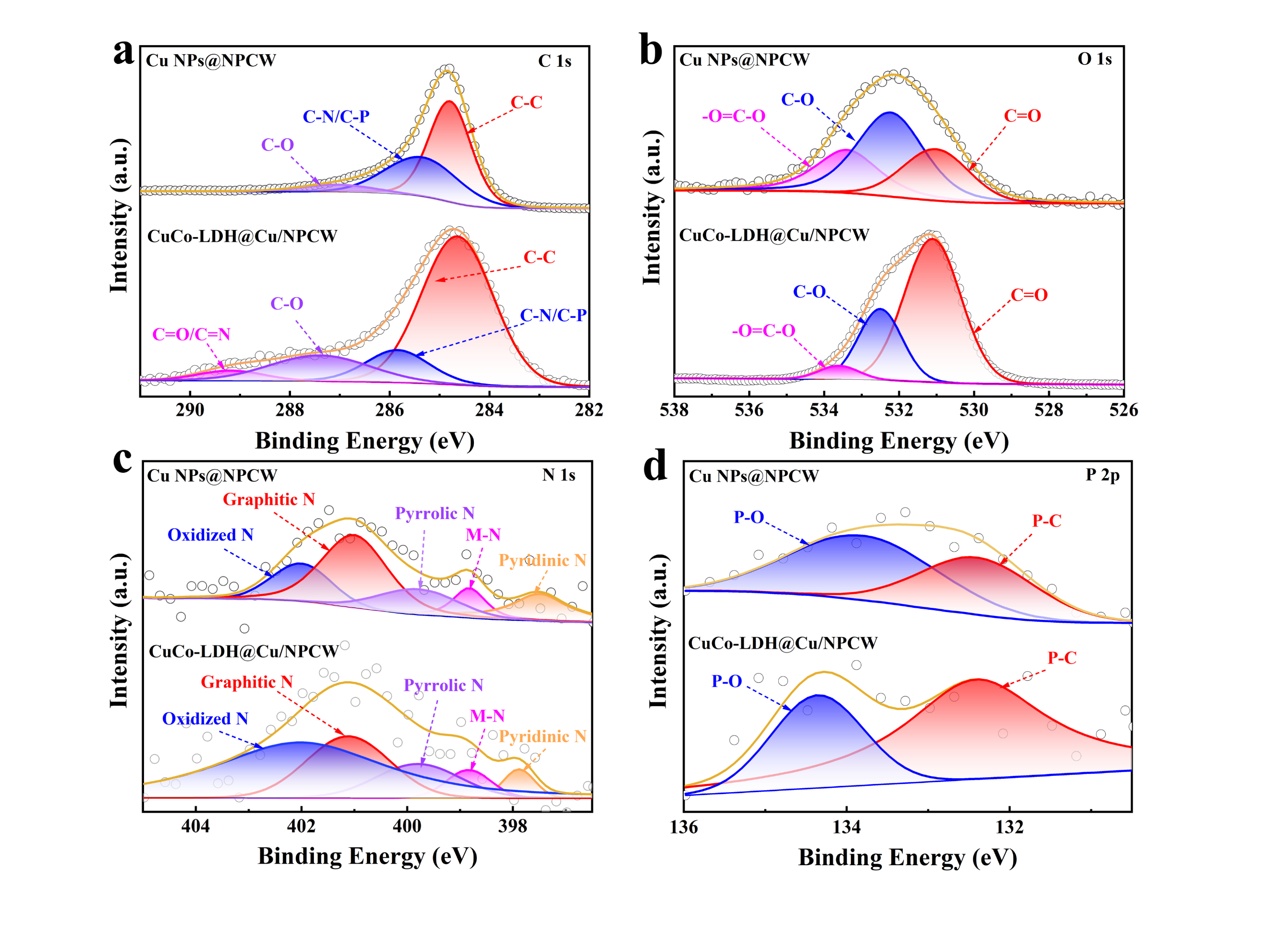


**Fig. S11** XPS spectra of (a) C 1s, (b) O 1s, (c) N 1s, and (d) P 2p of Cu NPs@NPCW and CuCo-LDH@Cu/NPCW electrodes.


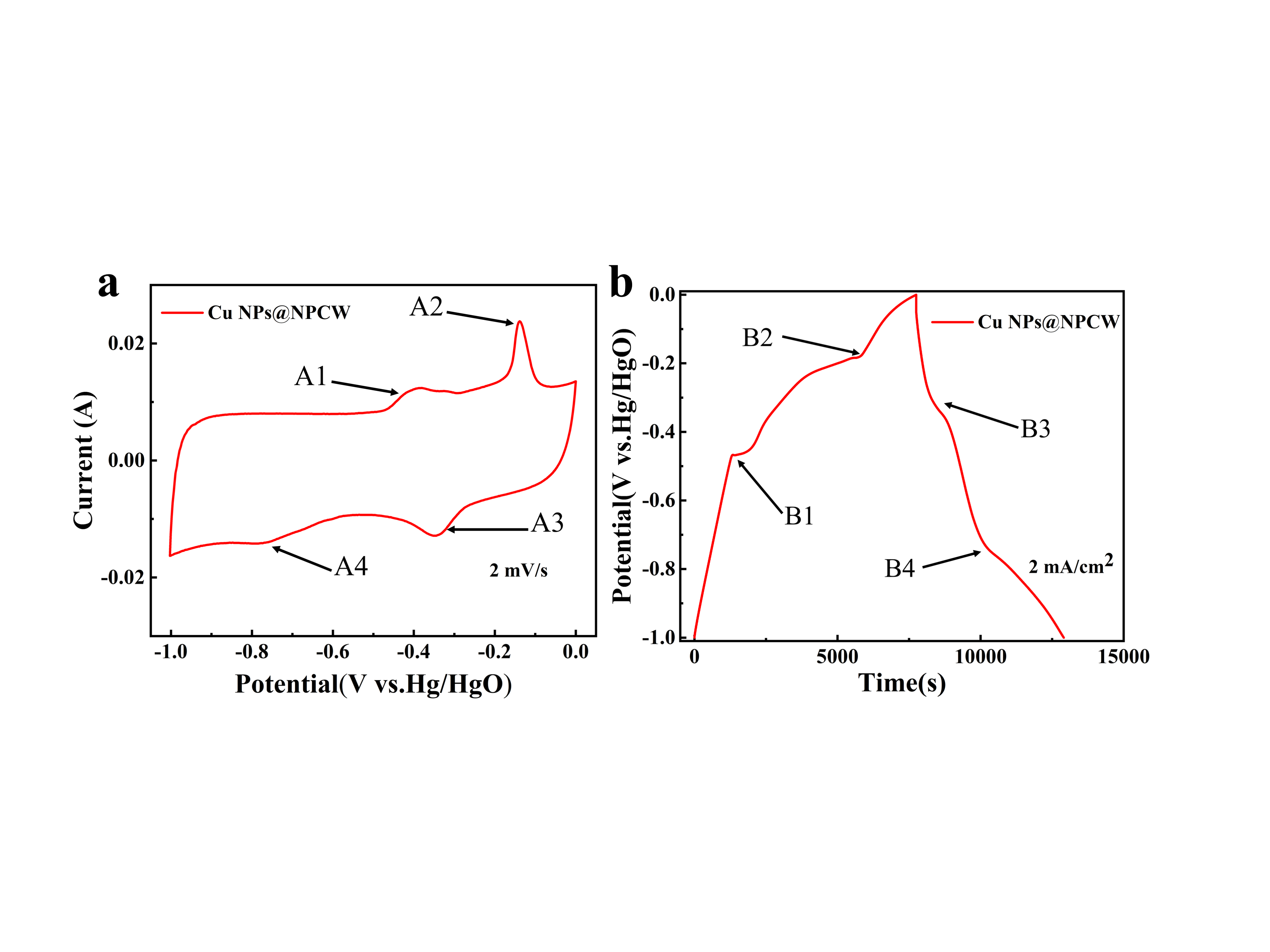


**Fig. S12** (a) CV curves at 2 mV s^-1^and (b) GCD curves at 2 mA cm^-2^ of Cu NPs@NPCW.

The peaks appearing in the CV curves are generated by the redox reaction of Cu NPs in KOH solution. As shown in Fig. S11a, during forward scanning, the first oxidation peak, A1, can be attributed to the oxidation of Cu to Cu_2_O (Eq. (1)); the second oxidation peak, A2, can be attributed to the oxidation of Cu_2_O to CuO (Eq. (1)). During the reverse scanning process, the first reduction peak, A3, can be attributed to the reduction of CuO to Cu_2_O; the second reduction peak, A4, can be attributed to the reduction of Cu_2_O to Cu^[3, 4]^. As shown in Fig. S11b, the voltage plateau exhibited by the Cu NPs@NPCW sample in the GCD curve is coincident with the redox peak in the CV curve.

The reaction equation is as follows:

$2Cu+ 2{OH}^{-} \leftrightarrow{Cu}_{2}O +H_{2} O + 2e^{-}$ (1)

${Cu}_{2}O+ 2{OH}^{-} \leftrightarrow2CuO +H_{2} O + 2e^{-}$ (2)


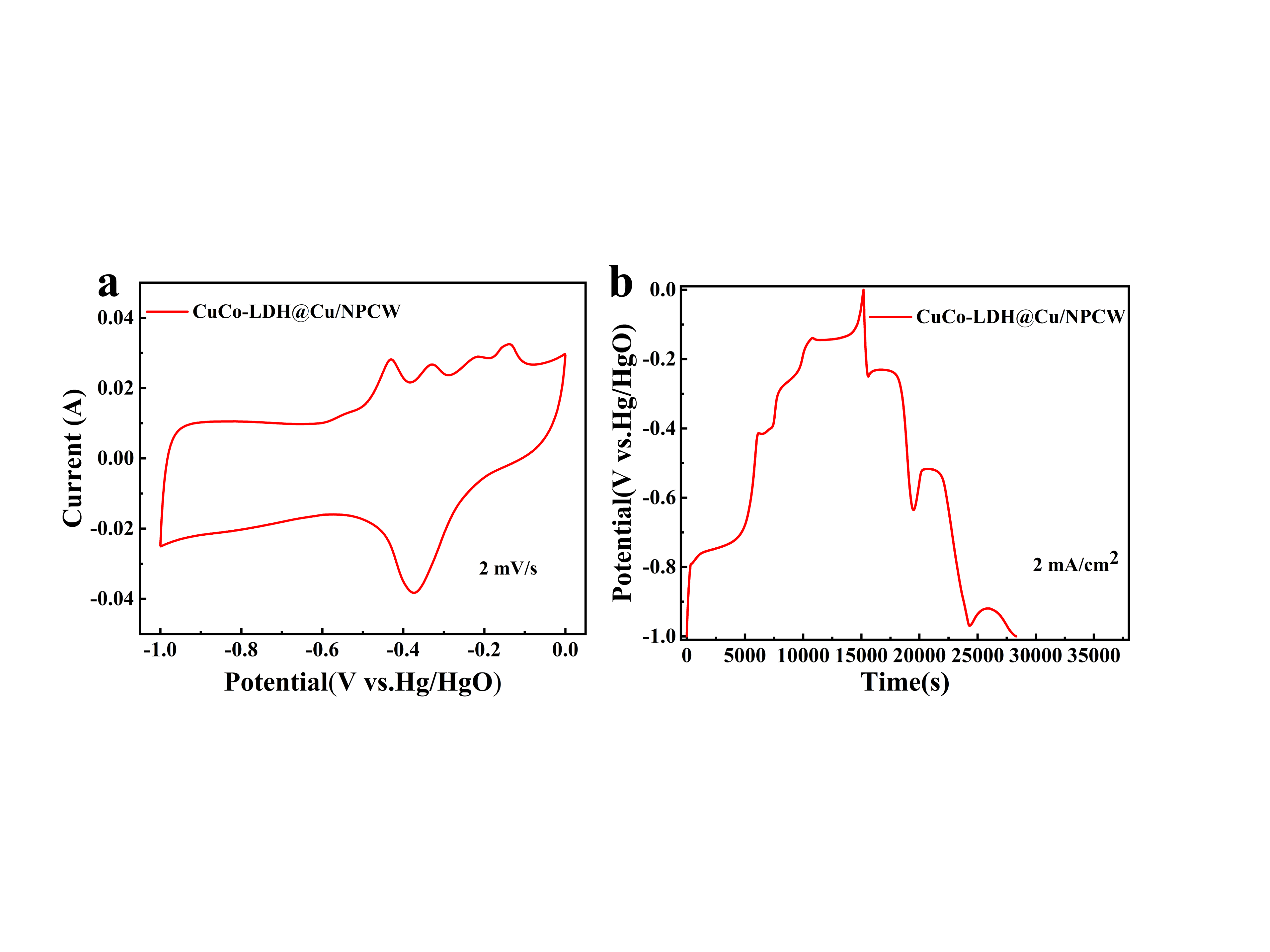


**Fig. S13** (a) CV curves at 2 mV s^-1^and (b) GCD curves at 2 mA cm^-2^ of CuCo-LDH@Cu/NPCW.


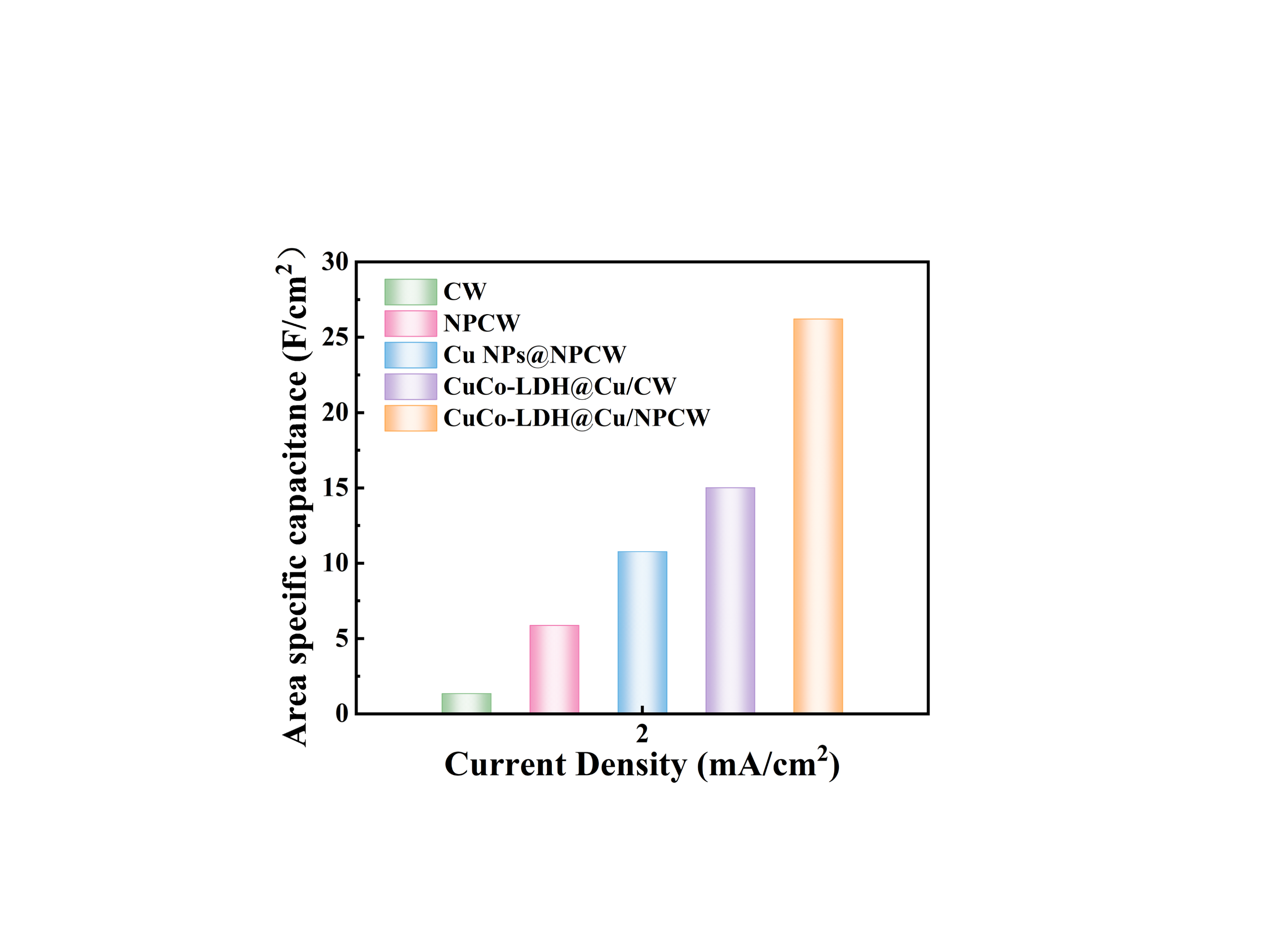


**Fig. S14** Specific capacitance of CW, NPCW, Cu NPs@NPCW, CuCo-LDH@Cu/CW, and CuCo-LDH@Cu/NPCW electrodes at 2 mA cm^-2^.


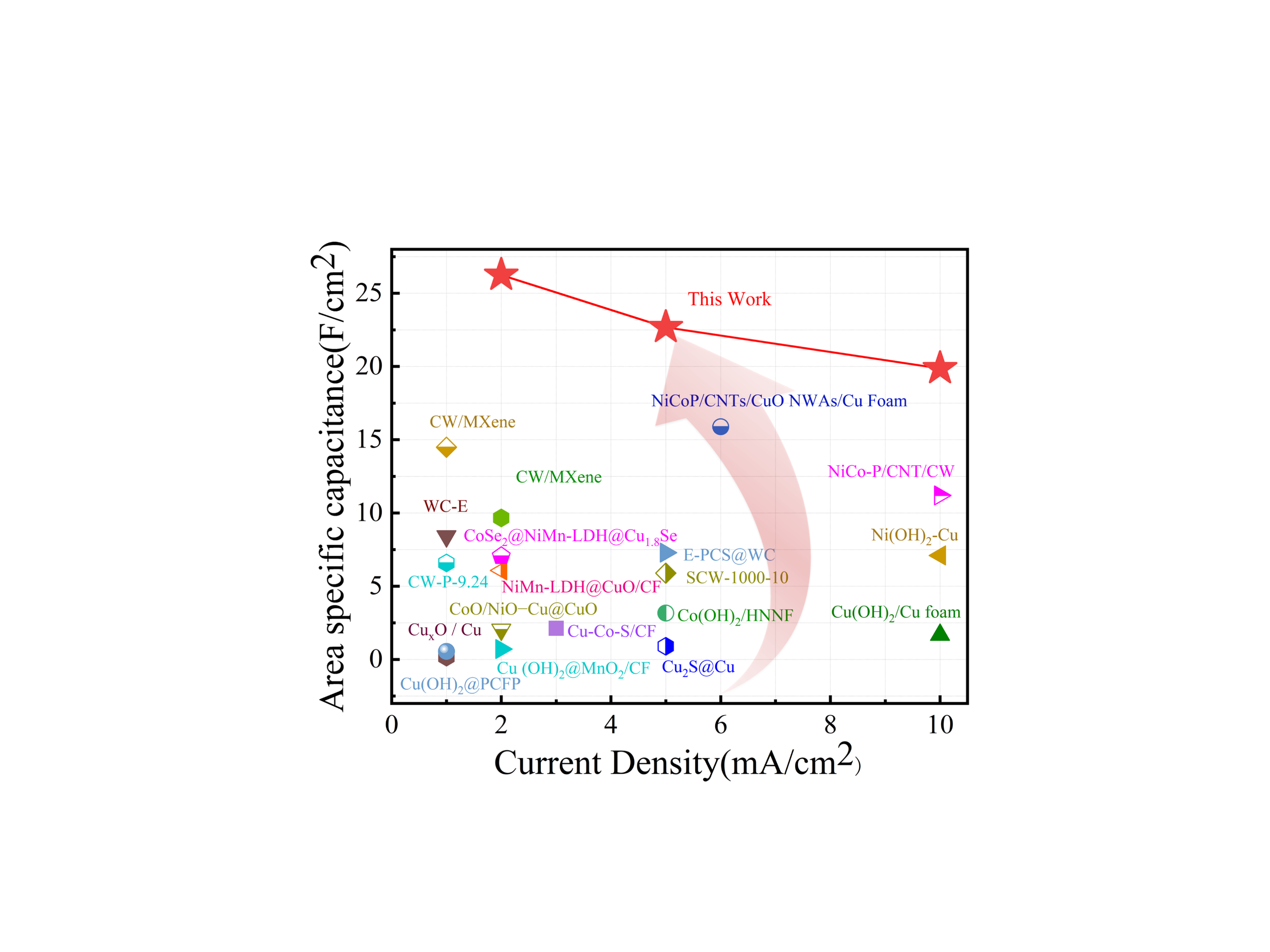


**Fig. S15** The Ragone plot of CuCo-LDH@Cu/NPCW for the comparison with the reported carbon-based/CuCo-based electrodes.^[5-20]^


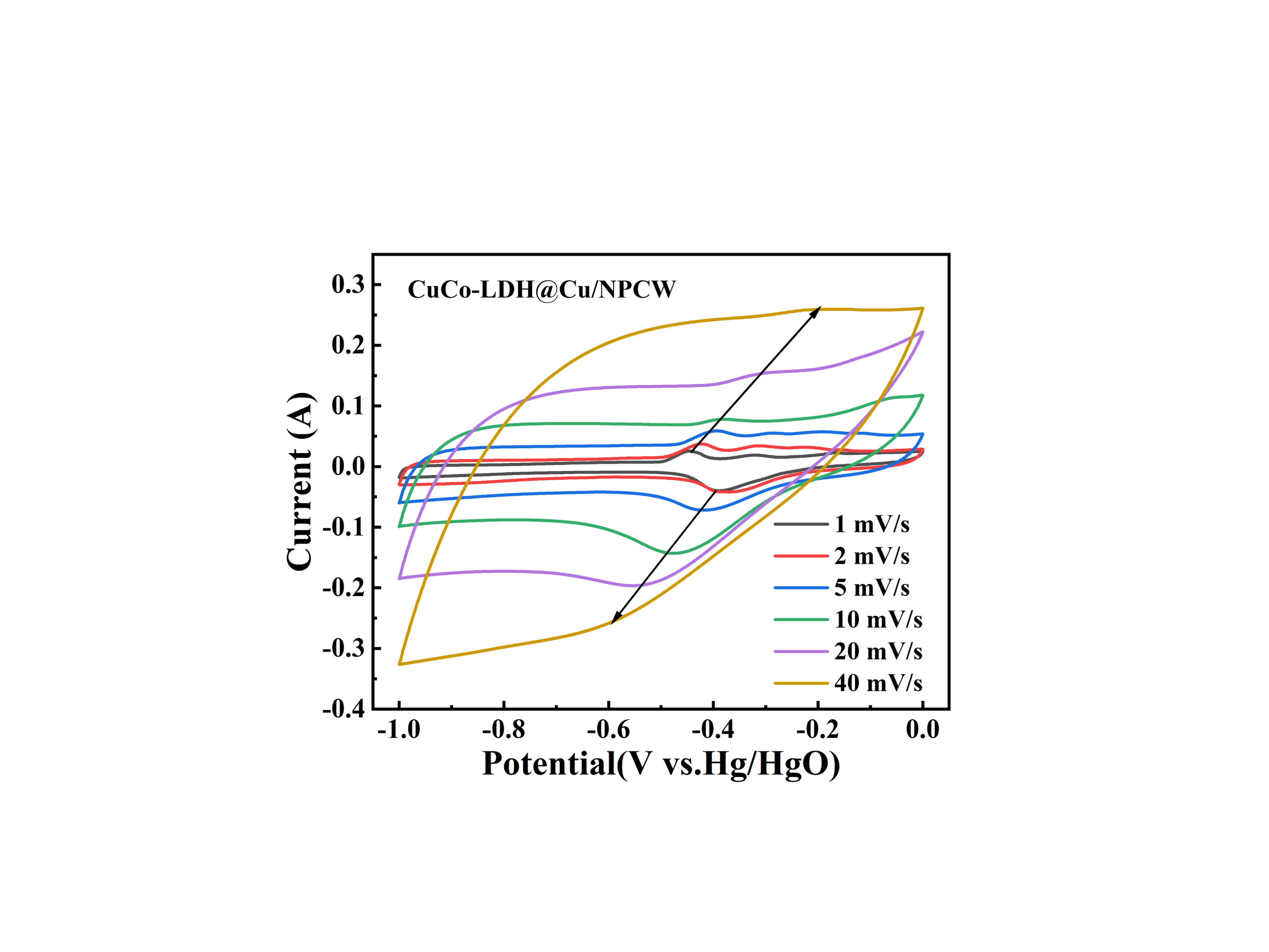


**Fig. S16** CV curves of CuCo-LDH@Cu/NPCW at a scanning rate of 1 mV s^−1^ to 40 mV s^−1^.

^
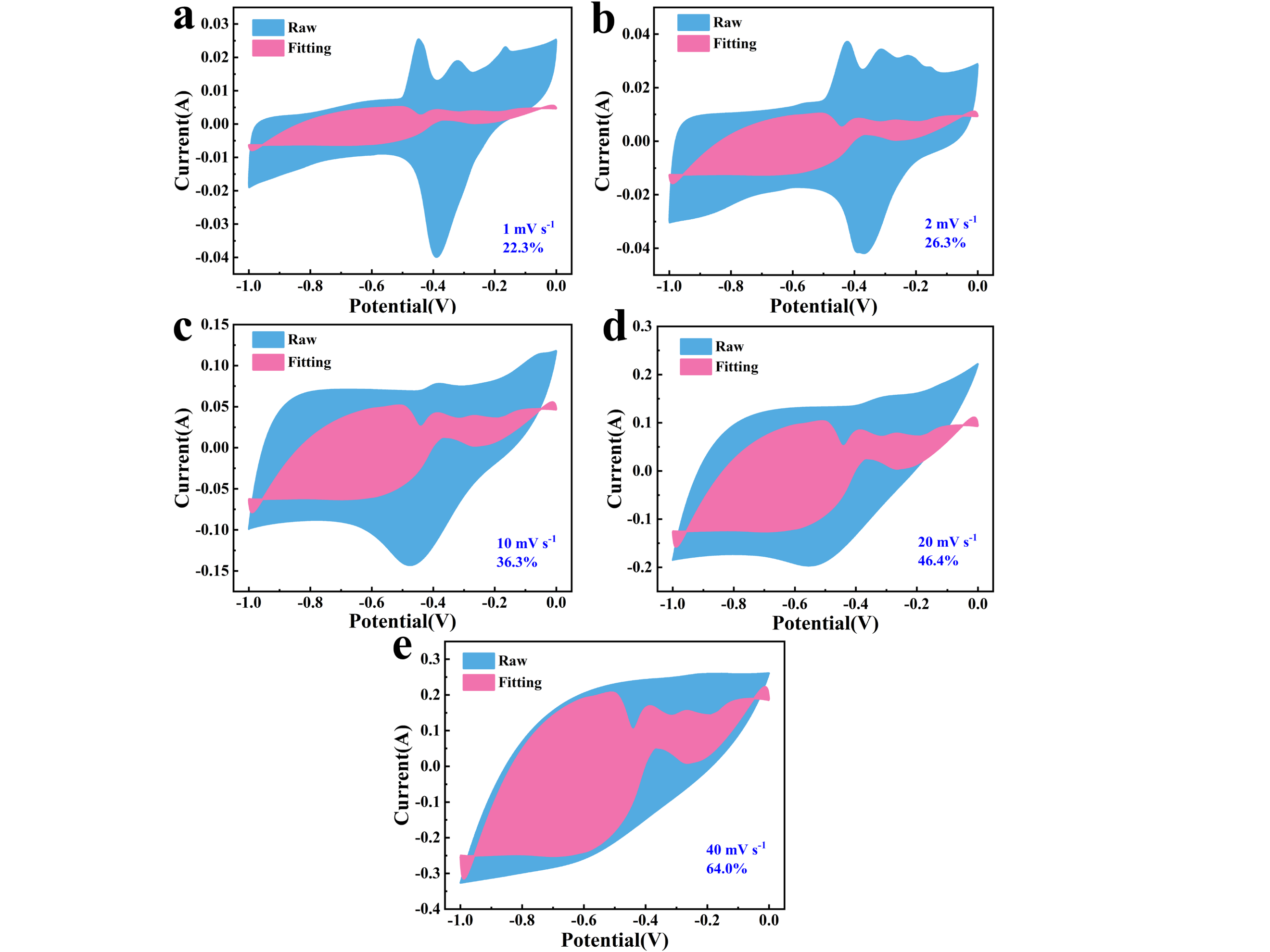
^

**Fig. S17** (a-e) The ratio of capacitance contribution of CuCo-LDH@Cu/NPCW to total capacitance at scanning rates of 1, 2, 10, 20, and 40 mV s^−1^.


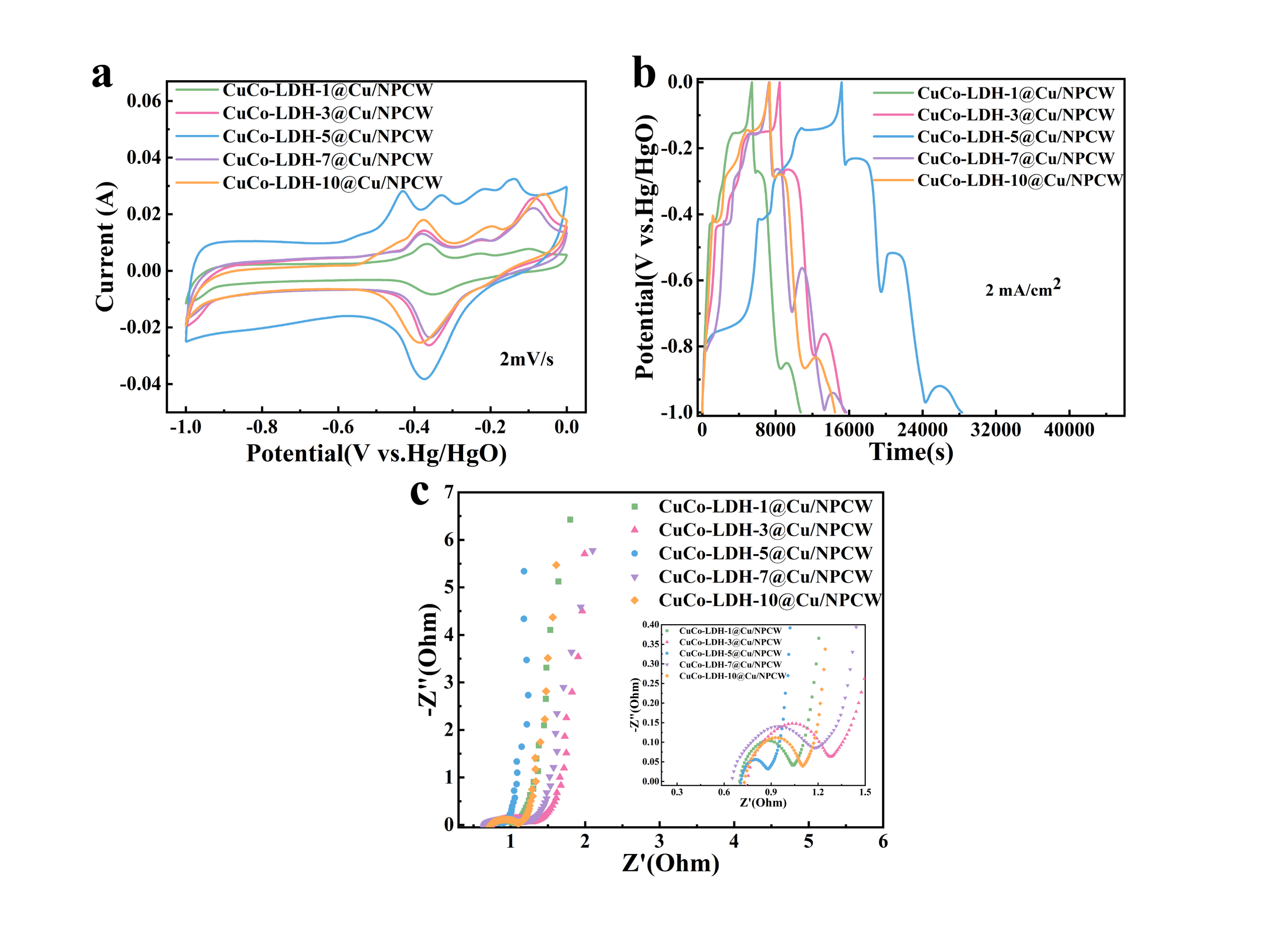


**Fig. S18** Electrochemical properties of CuCo-LDH-y@Cu/NPCW prepared with different Co^2+^: (a) CV curve at a scan rate of 2 mV s^-1^; (b) GCD curve at a current density of 2 mA cm^-2^; (c) EIS spectrum.

^
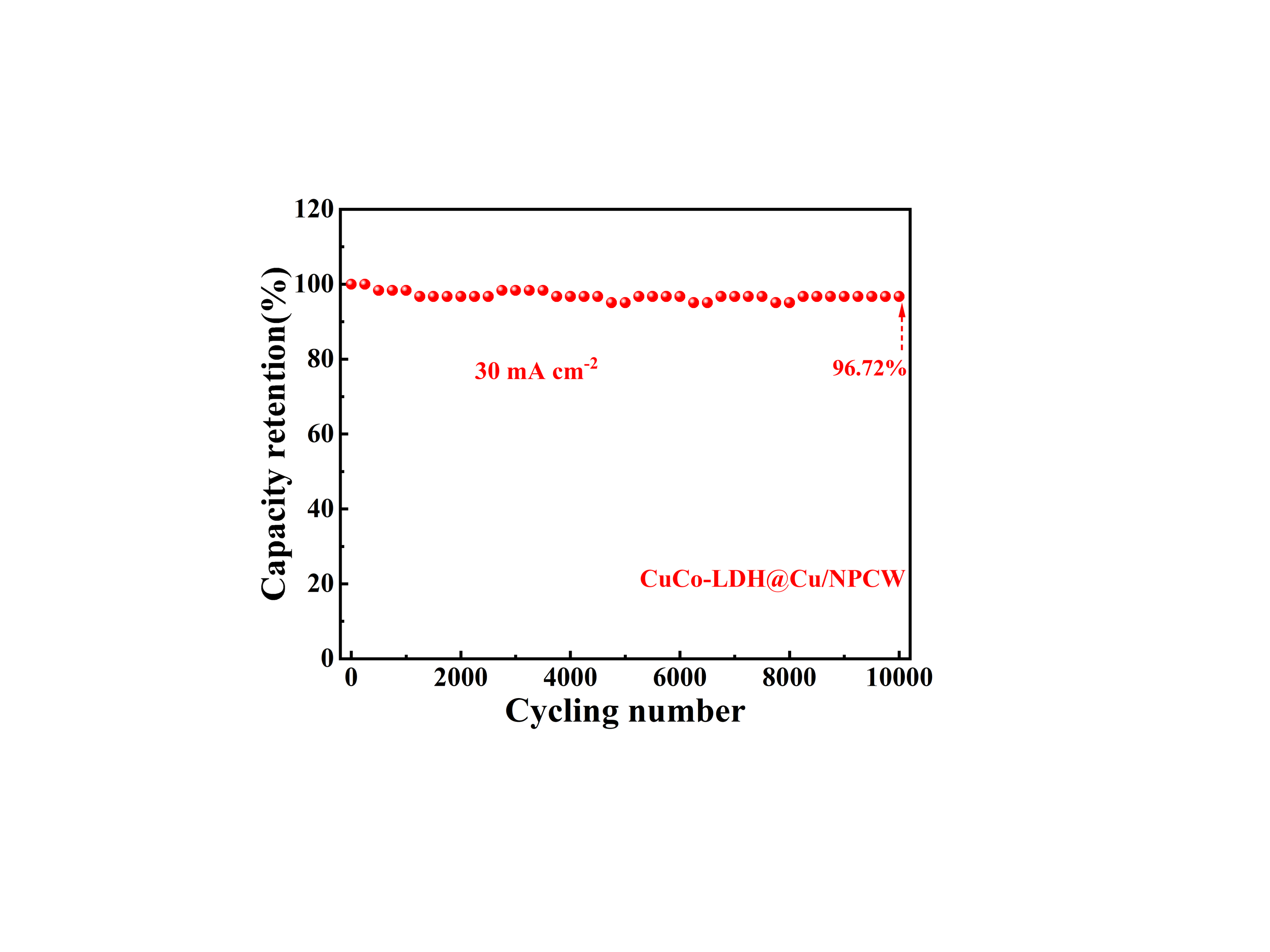
^

**Fig. S19** Stability of CuCo-LDH@Cu/NPCW for 10,000 cycles at 30 mA cm^-2^.

^
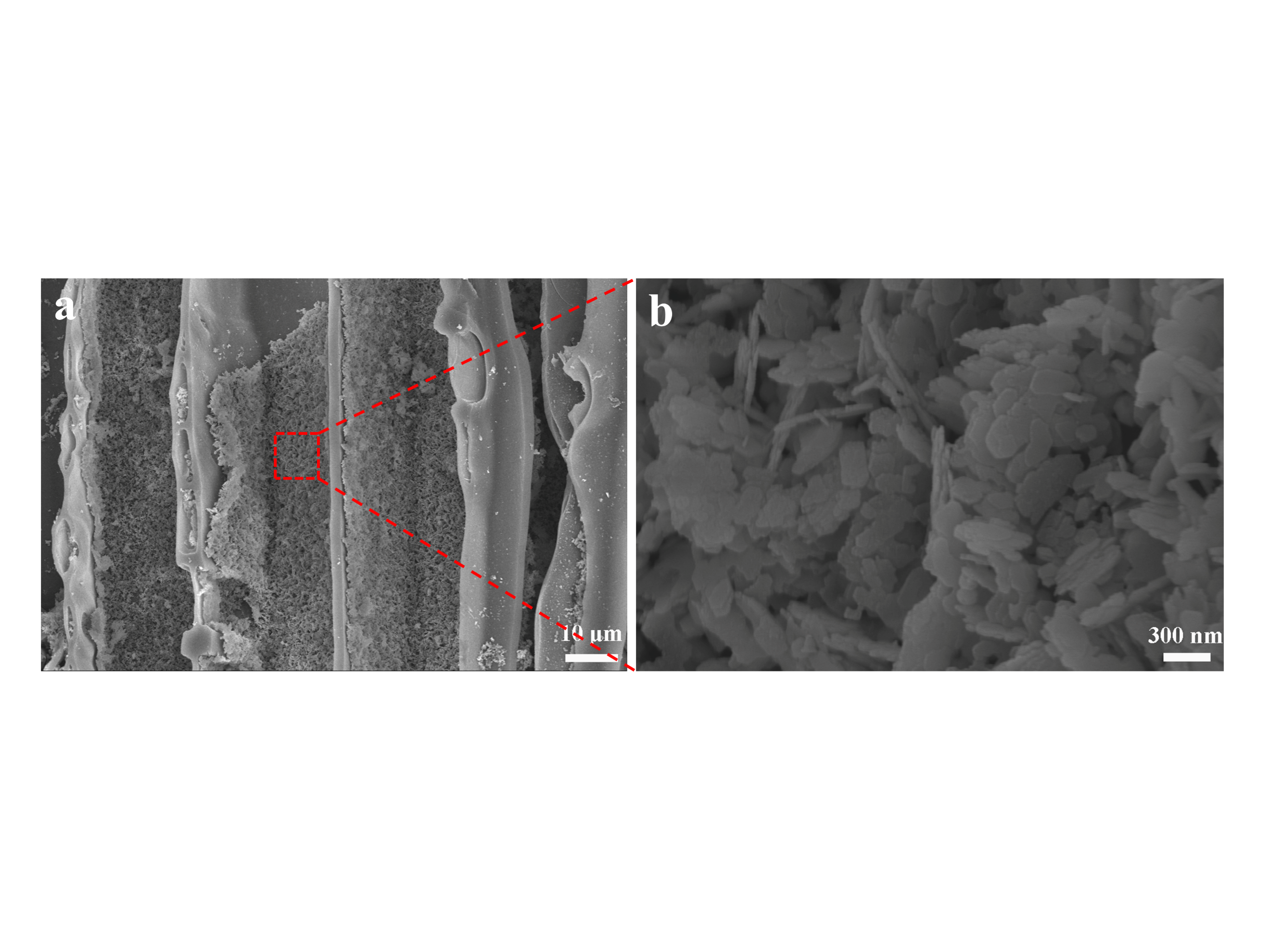
^

**Fig. S20** (a, b) SEM images of CuCo-LDH@Cu/NPCW after the 10,000 cycles stability test at a current density of 30 mA cm^‒1^.

^
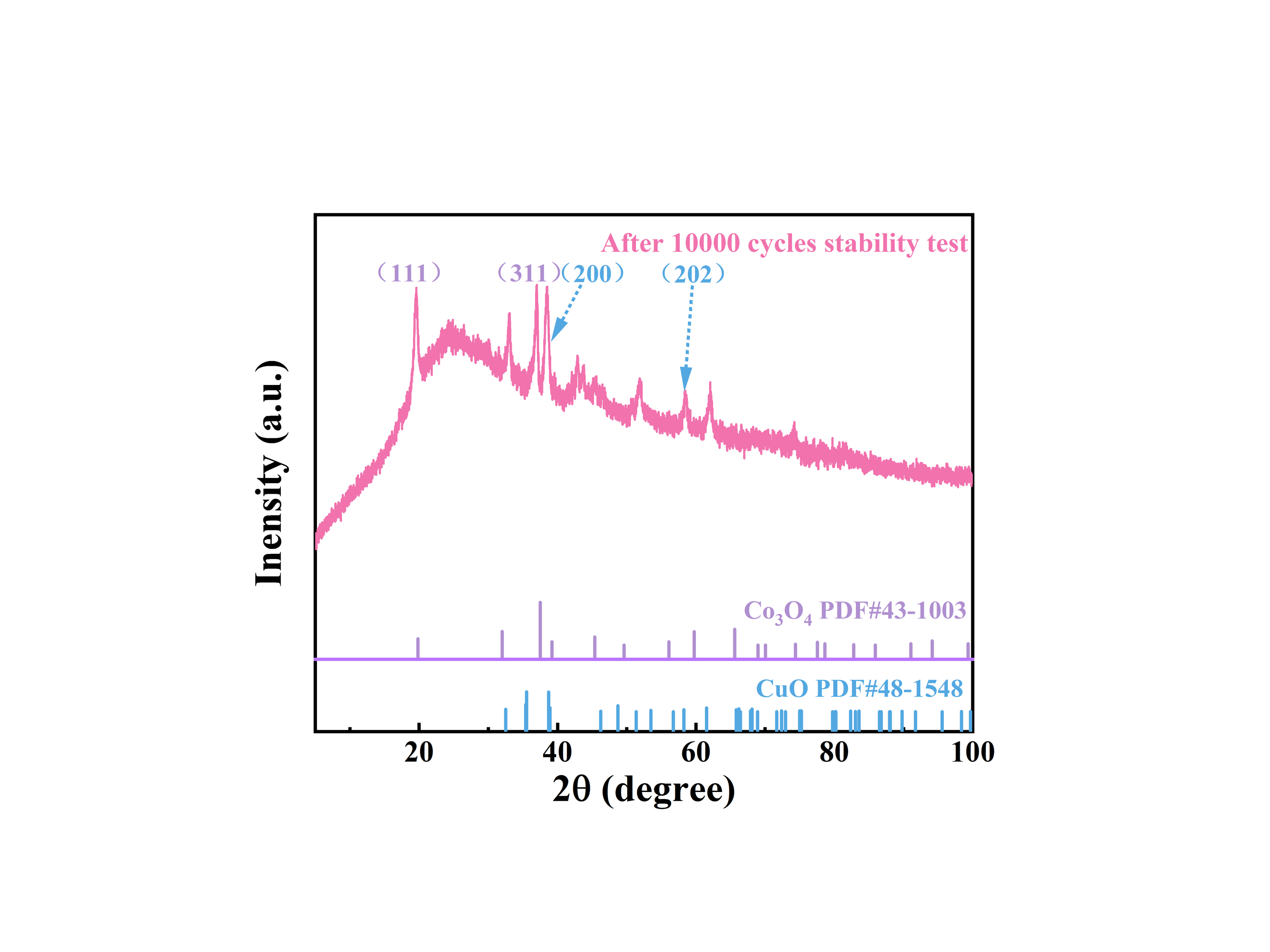
^

**Fig. S21** XRD patterns of CuCo-LDH@Cu/NPCW after the 10,000 cycles stability test at a current density of 30 mA cm^‒1^.

^
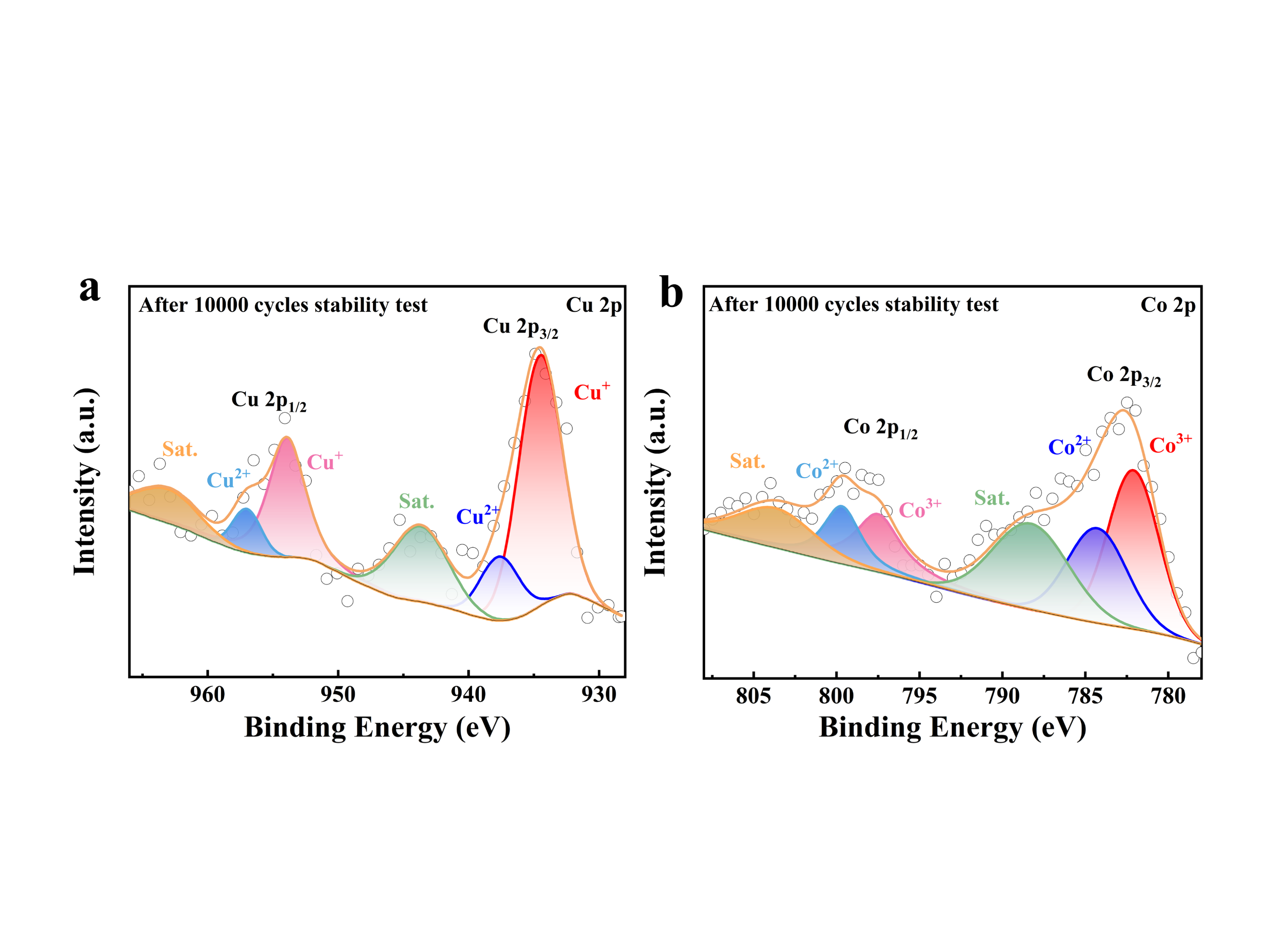
^

**Fig. S22** (a, b) Cu 2*p* and Co 2*p* high-resolution XPS spectra of CuCo-LDH@Cu/NPCW after the 10,000 cycles stability test at a current density of 30 mA cm^‒1^.

^
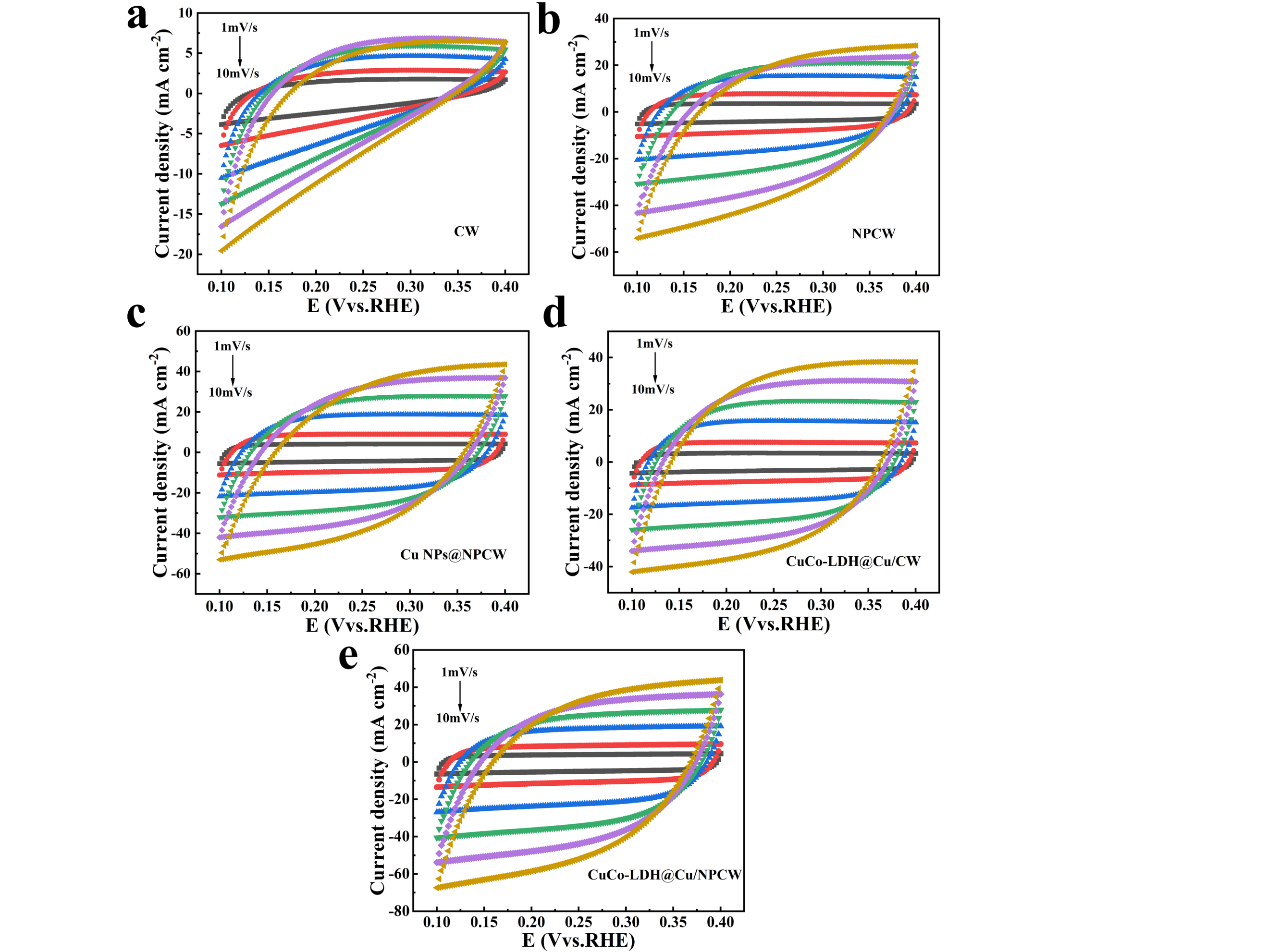
^

**Fig. S23** CV curves of (a) CW, (b) NPCW, (c) Cu NPs@NPCW, (d) CuCo-LDH@Cu/CW, and (e) CuCo-LDH@Cu/NPCW, the scan rates ranged from 1 to 10 mV s^‒1^, the scanning potential range was from 0.10 to 0.40 V.

^
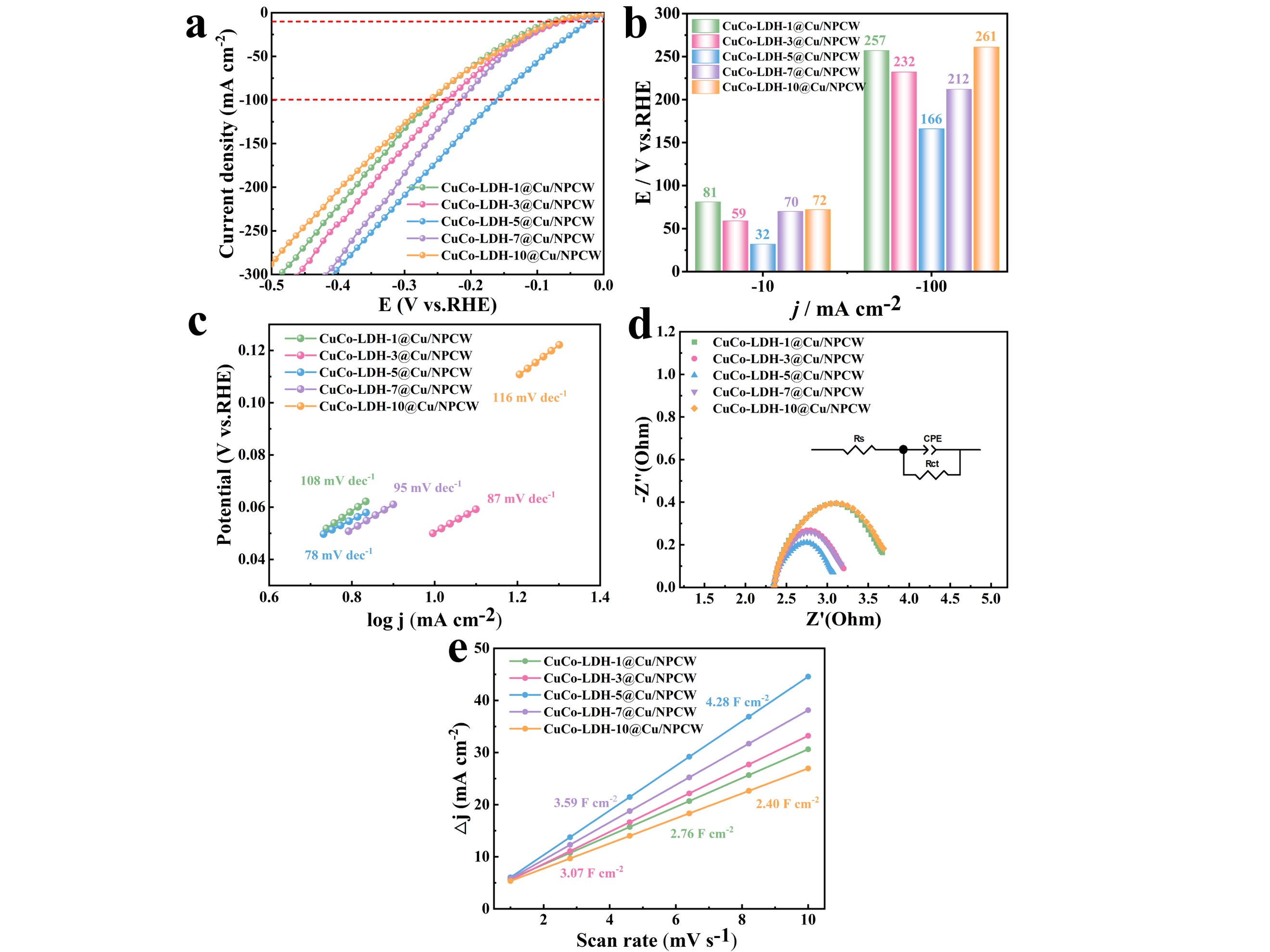
^

**Fig. S24** HER properties of CuCo-LDH-y@Cu/NPCW prepared with different Co^2+^:(a) The HER polarization curves of electrocatalysts; (b) Comparison of the overpotentials required at 10 mA cm^-2^ and 100 mA cm^-2^ of different catalysts; (c) Tafel plots; (d) EIS spectra; (e) Cdl values.


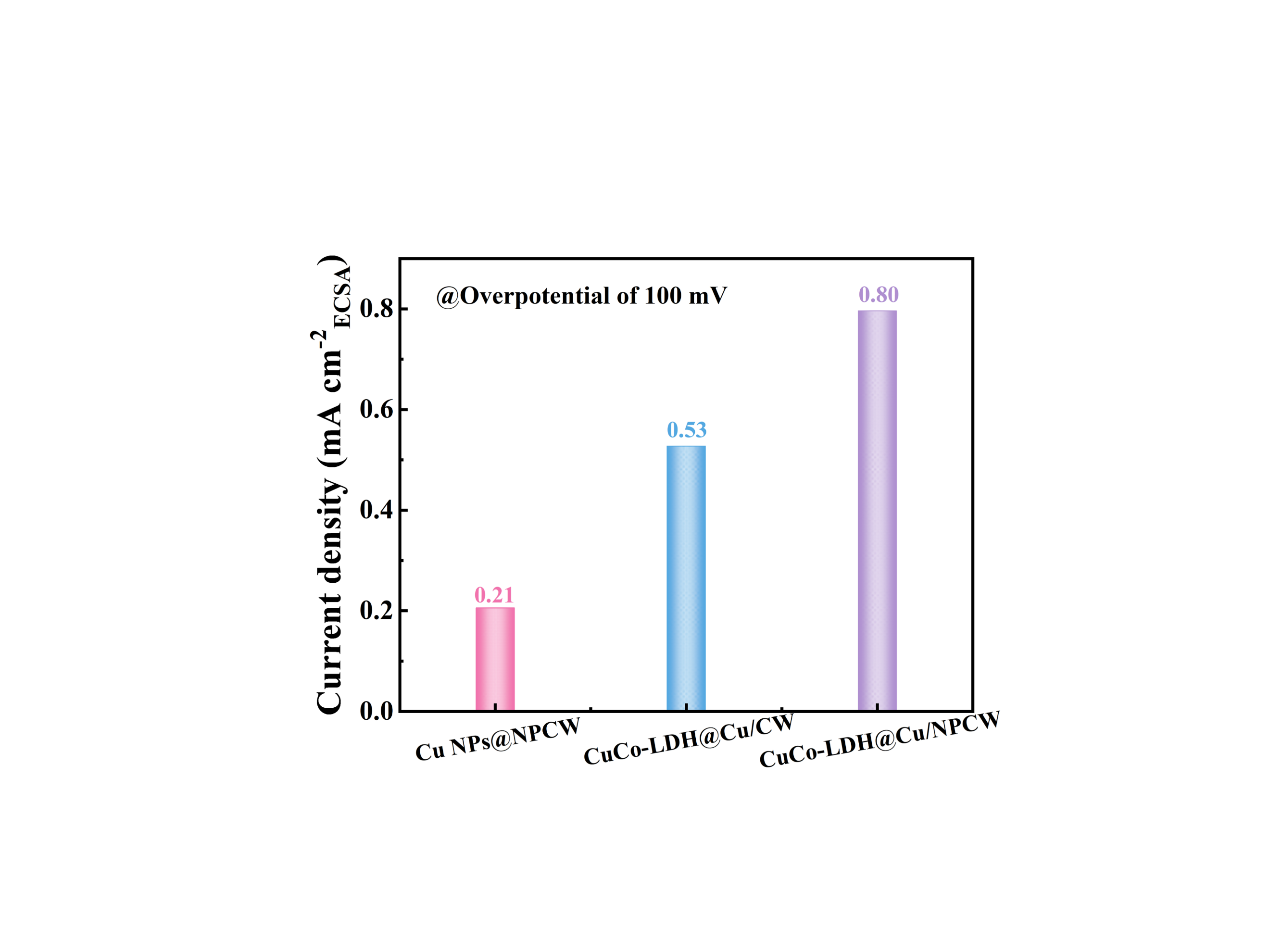


**Fig. S25** The normalized electrochemically active area of Cu NPs@NPCW, CuCo-LDH@Cu/CW, and CuCo-LDH@Cu/NPCW at a 100 mV overpotential.

^
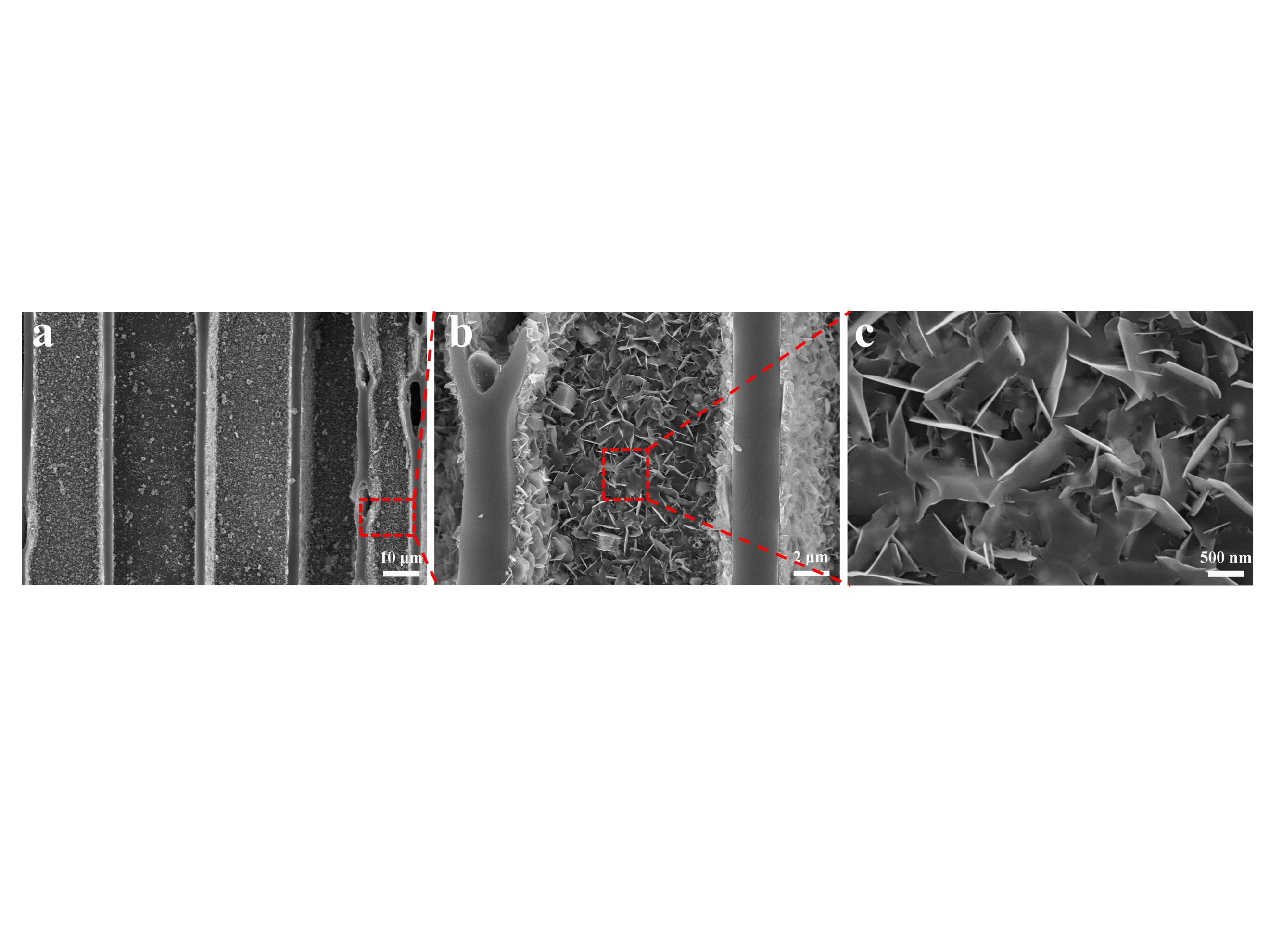
^

**Fig. S26** (a-c) SEM images of CuCo-LDH@Cu/NPCW after the HER CP stability test.


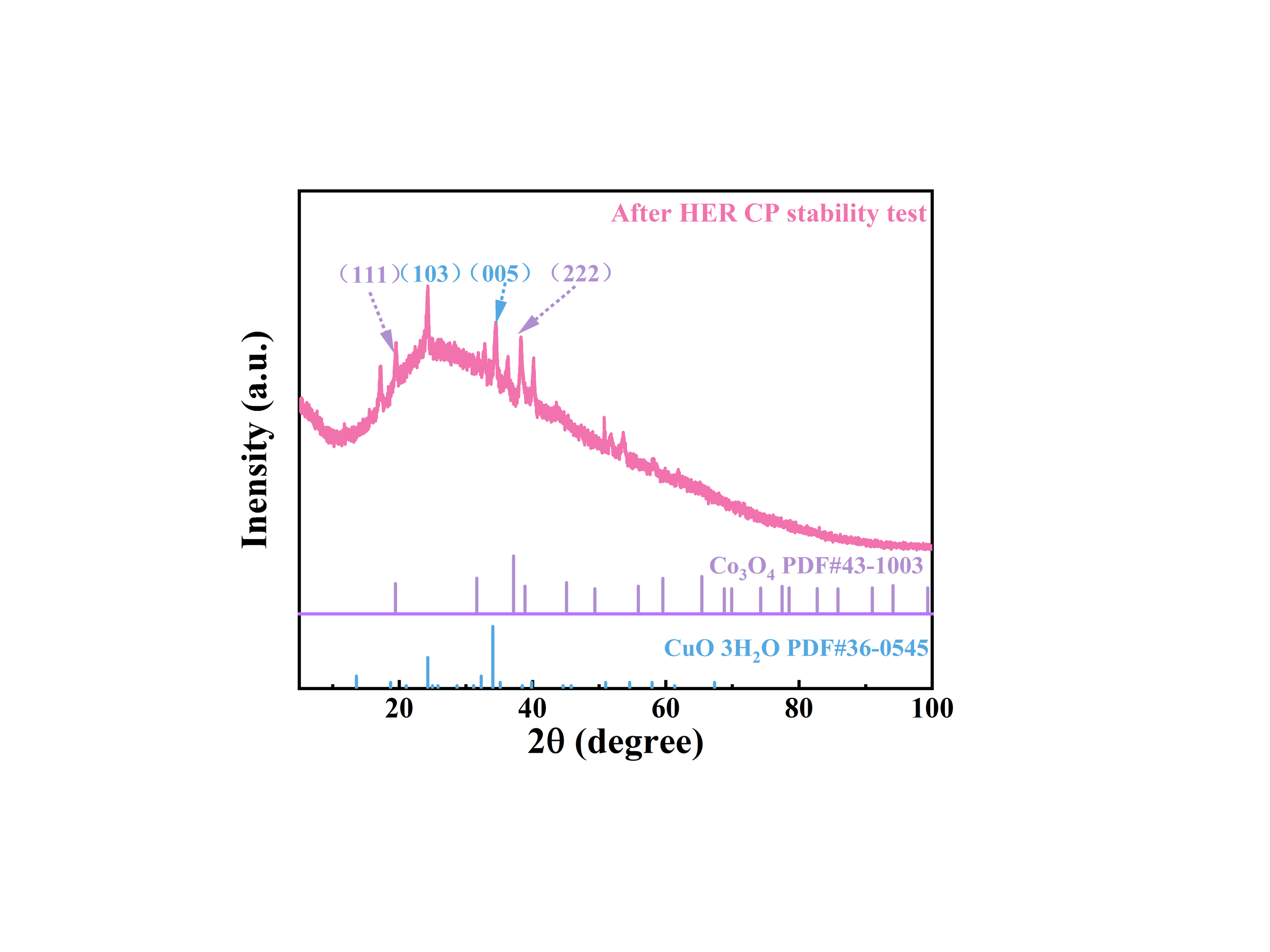


**Fig. S27** XRD patterns of CuCo-LDH@Cu/NPCW after the HER CP stability test.


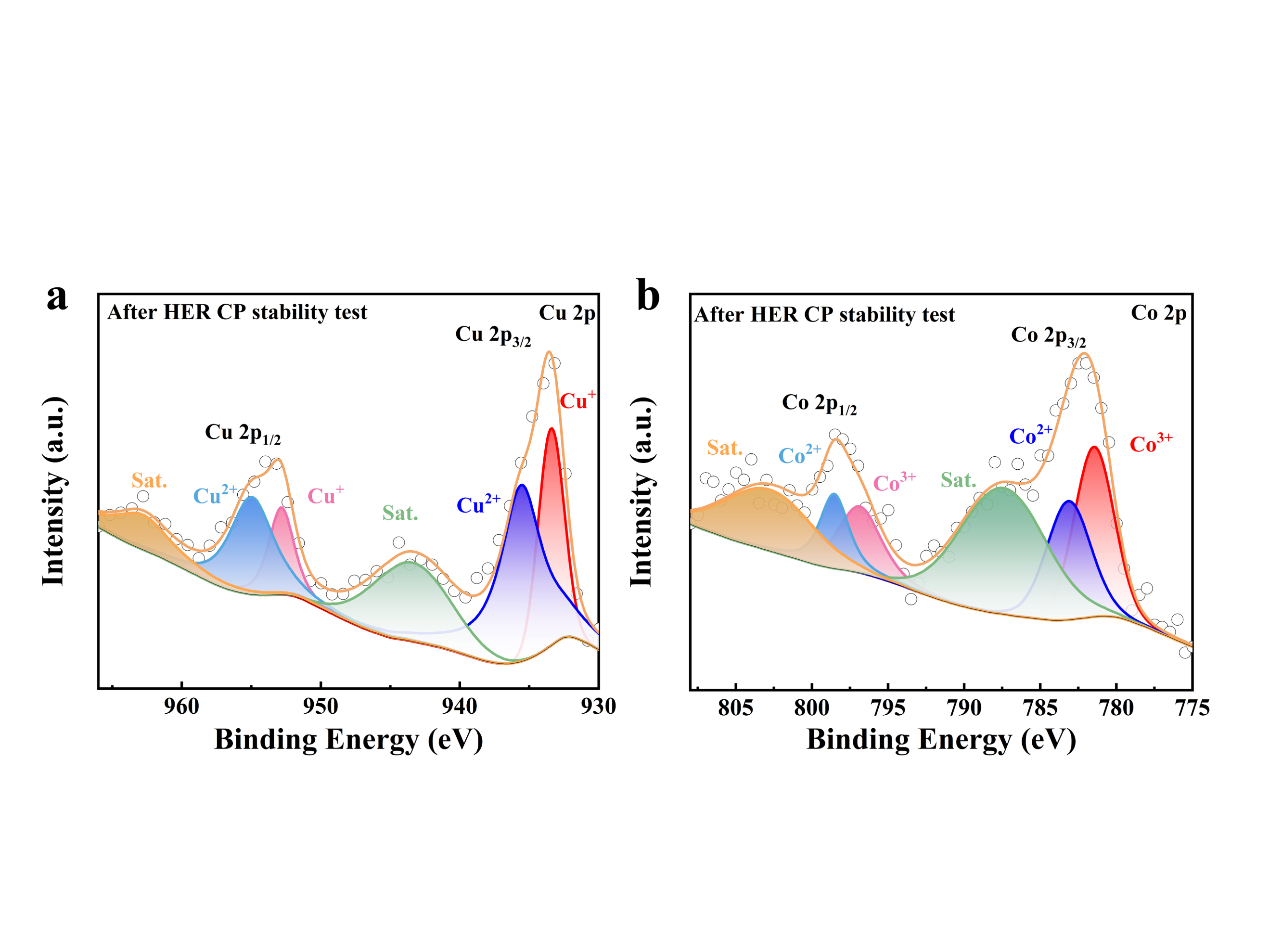


**Fig. S28** (a, b) Cu 2*p* and Co 2*p* high-resolution XPS spectra of CuCo-LDH@Cu/NPCW after the HER CP stability test.

**3. Supplementary Tables**

**Table S1** Elemental Cu/Co content of samples obtained from ICP-OES.

| **Sample** | **Cu (wt%)** | **Co (wt%)** |
| --- | --- | --- |
| Cu NPs@NPCW | 26.94 | - |
| CuCo-LDH@Cu/CW | 10.03 | 4.29 |
| CuCo-LDH@Cu/NPCW | 16.89 | 8.40 |

**Table S2** Pore properties of CW, NPCW, Cu NPs@NPCW, CuCo-LDH@Cu/CW, and CuCo-LDH@Cu/NPCW.

| **Sample** | **Pore size**  **(nm)** | **S_BET_**  **(m^2^/g)** | **V_total_**  **(cm^3^/g)** |
| --- | --- | --- | --- |
| CW | 1.11 | 335.75 | 0.14 |
| NPCW | 0.49 | 1678.72 | 0.24 |
| Cu NPs@NPCW | 0.67 | 1223.38 | 0.21 |
| CuCo-LDH@Cu/CW | 1.07 | 1035.69 | 0.28 |
| CuCo-LDH@Cu/NPCW | 0.63 | 1419.22 | 0.46 |

**Table S3** XPS analysis of Cu NPs@NPCW and CuCo-LDH@Cu/NPCW.

| **Sample** | **C(at.%)** | **O(at.%)** | **N(at.%)** | **P(at.%)** | **Cu(at.%)** | **Co(at.%)** |
| --- | --- | --- | --- | --- | --- | --- |
| Cu NPs@NPCW | 89.74 | 7.32 | 2.13 | 0.64 | 0.16 | - |
| CuCo-LDH@Cu/NPCW | 75.44 | 18.45 | 2.51 | 1.21 | 1.36 | 1.04 |

**Table S4** Performance comparison of supercapacitors with different carbon-based/CuCo-based electrodes.

| **Sample** | **Current density (mA cm^-2^)** | **Area specific capacitance**  **(F cm^-2^)** | **Mass specific capacitance**  **(F g^-1^)** | | **Reference** |
| --- | --- | --- | --- | --- | --- |
| **CuCo-LDH@Cu/NPCW** | **2**  **5**  **10** | **26.24**  **22.67**  **19.87** | **749.71**  **647.71**  **567.71** | | **This Work** |
| CW/MXene | 1 | 4.24 | | 80.61 | ^[5]^ |
| CW-P-9.24 | 1 | 6.59 | | 384.00 | ^[7]^ |
| SCW-1000-10 | 1 | 6.63 | | 143.60 | ^[16]^ |
| CRTW-3 | 1 | 6.11 | | 413.60 | ^[21]^ |
| WC@MnO_2_ -20 | 1 | 3.62 | | 116.00 | ^[22]^ |
| a-WC-Mn | 1 | 11.90 | | - | ^[23]^ |
| CWC | 1 | 3.04 | | 118.00 | ^[24]^ |
| WC-E-100-48 | 1 | 8.41 | | - | ^[6]^ |
| GPCN-2 | 1 | 10.2 | | 508.00 | ^[25]^ |
| HWC-3M | 1 | 3.39 | | 138.30 | ^[26]^ |
| ACC@BCN | 1 | 1.02 | | - | ^[27]^ |
| CW-TA-60 | 0.5 | 1.32 | | - | ^[28]^ |
| CW | 5 | 1.77 | | 32.70 | ^[16]^ |
| ACW | 5 | 5.60 | | 114.70 | ^[16]^ |
| E-PCS@WC | 5 | 7.29 | | - | ^[15]^ |
| PCS@WC | 5 | 3.14 | | - | ^[15]^ |
| Cu-HHB | 0.4 | 0.11 | | - | ^[29]^ |
| PrGO/Cu(OH)_2_ /Cu@CF | 0.5 | 0.72 | | - | ^[30]^ |
| 1T-MoS_2_/Cu(OH)_2_@CFP | 1 | 1.12 | | - | ^[31]^ |
| CuO@NiCoZn-LDH | 1 | 1.82 | | - | ^[32]^ |
| Co(OH)_2_@CW | 1 | 3.72 | | 100.30 | ^[33]^ |
| Co(OH)_2_ /PBA/CC | 1 | 1.18 | | - | ^[34]^ |
| NiMn-LDH@CuO/CF | 2 | 6.08 | | 2430.00 | ^[11]^ |
| CuCo-LDH-S/ZnS-QDs | 2 | 7.82 | | - | ^[35]^ |
| CoSe_2_@NiMn-LDH@Cu_1.8_Se | 2 | 7.06 | | - | ^[10]^ |
| Cu NPs@NCW | 2 | 9.66 | | 284.14 | ^[3]^ |
| CuCo_2_S_4_@MnO_2_ | 3 | 14.90 | |  | ^[36]^ |
| CuCo LDH@Ni_3_S_2_ | 5 | 11.24 | |  | ^[37]^ |
| NiCoP/CNTs/CuO | 6 | 15.88 | | - | ^[38]^ |
| NiCo-P/CNT0.25/CW | 10 | 11.20 | | 230.00 | ^[19]^ |

**Table S5** Pore properties of CW, NPCW, Cu NPs@NPCW, CuCo-LDH@Cu/CW, and CuCo-LDH@Cu/NPCW.

| **Electrode** | **Rs (Ω)** | **Rct (Ω)** |
| --- | --- | --- |
| CW | 0.83 | 0.43 |
| NPCW | 0.75 | 0.20 |
| Cu NPs@NPCW | 0.72 | 0.13 |
| CuCo-LDH@Cu/CW | 0.78 | 0.28 |
| CuCo-LDH@Cu/NPCW | 0.70 | 0.17 |

**Table S6** Comparison of the HER activity of CuCo-LDH@Cu/NPCW with the reported catalysts.

| **Catalysts** | **Tafel slope**  **(mV dec^−1^)** | **Potential**  **@*j* (mV)** | **KOH**  **(M)** | **Reference** |
| --- | --- | --- | --- | --- |
| **CuCo-LDH@Cu/NPCW** | **78.0** | **32@−10** | **1.0** | **This Work** |
| ZnMnSP/NDC | 76.5 | 178@−10 | 1.0 | ^[39]^ |
| EM-FeNiP@HCNT | 77.4 | 294@−10 | 1.0 | ^[40]^ |
| N，P-Mo_2_C/NPC | 58.2 | 284@−10 | 1.0 | ^[41]^ |
| N，P- NiMoO_x_/NF | 95.4 | 57@−10 | 1.0 | ^[42]^ |
| MoP-MoS_2_/HCSs | 47.8 | 71@−10 | 1.0 | ^[43]^ |
| Ru@N-P-C | 115.0 | 45@−10 | 1.0 | ^[44]^ |
| Ni-Mo_2_C/NPC | 64.0 | 244@−10 | 1.0 | ^[45]^ |
| CoMoP@N, P-C | 76.8 | 179@−10 | 1.0 | ^[46]^ |
| CuO@Co_3_O_4_ | 48.0 | 82@−10 | 1.0 | ^[47]^ |
| Co/N-NiMo_3_S_4_ | 69.0 | 78@−10 | 1.0 | ^[48]^ |
| Cu/N-NiMo_3_S_4_ | 88.0 | 108@−10 | 1.0 | ^[48]^ |
| Ni_1.8_Cu_0.2_-P | 70.0 | 78@−10 | 1.0 | ^[49]^ |
| Co(OH)_2_/1T-MoS_2_ | 94.0 | 151@−10 | 1.0 | ^[50]^ |
| CuCo_2_S_4_/NF | 41.8 | 142@−10 | 1.0 | ^[51]^ |
| Ni_3_ZnC_0.7_@CoNiCuFe -NC | 94.3 | 97@−10 | 1.0 | ^[52]^ |
| Cu-Co-P/60@NF | 81.0 | 102@−10 | 1.0 | ^[53]^ |
| Co SAs-Co NPs/NCFs | 83.2 | 205@−10 | 1.0 | ^[54]^ |
| Co,Ni-MoTe_2_ | 87.3 | 82@−10 | 1.0 | ^[55]^ |
| Co(OH)_2_/Fe_7_Se_8_ | 116.0 | 183@−10 | 1.0 | ^[56]^ |
| Cu NPs@NCW | 95.0 | 100@−10 | 1.0 | ^[3]^ |
| N-NiCoPx/NCF | 30.0 | 23@−10 | 1.0 | ^[57]^ |
| Ce-Co/CoO@CNTs | 144.7 | 48@−10 | 1.0 | ^[58]^ |
| Co(OH)_2_/c-WN_1-x_/CNTs | 43.0 | 78@−10 | 1.0 | ^[59]^ |
| Co(OH)_2_@P-NiCo-LDH | 134.0 | 226@−10 | 1.0 | ^[60]^ |
| Cu-Ni_3_S_2_/Co_3_S_4_ | 50.4 | 79@−10 | 1.0 | ^[61]^ |
| CuCo_2_S_4_/N-rGO | 86.0 | 95@−10 | 1.0 | ^[62]^ |
| Cu_3_Se_2_@CoSe_2_−NiSe_2_/PNCF | 60.0 | 42@−10 | 1.0 | ^[63]^ |
| PtFeCoNiCuCr@HCS | 39.3 | 29@−10 | 1.0 | ^[64]^ |
| Co/CoP | 110.0 | 85@−10 | 1.0 | ^[65]^ |
| Co/CoP | 55.3 | 83@−10 | 1.0 | ^[66]^ |
| Cu(OH)_2_/Cu_2_S | 64.0 | 174@−10 | 1.0 | ^[67]^ |
| CoNiN/CMF | 92.0 | 37@−10 | 1.0 | ^[68]^ |
| NiCoP@NiCu LDH/NF | 314.7 | 158@−10 | 1.0 | ^[69]^ |
| NiCoP@NiCo LDH/NF | 150.1 | 154@−10 | 1.0 | ^[69]^ |

**References**

[1] H. Sun, Z. Luo, M. Chen, et al. Manipulating Trimetal Catalytic Activities for Efficient Urea Electrooxidation-Coupled Hydrogen Production at Ampere-Level Current Densities, ACS Nano. 18 (2024) 35654-35670.

[2] X. Liu, S. Zhang, J. Liang, et al. Protrusion-Rich Cu@NiRu Core@shell Nanotubes for Efficient Alkaline Hydrogen Evolution Electrocatalysis, Small. 18 (2022) e2202496.

[3] H. Hou, H. Huo, Y. Yu, et al. In situ growth of copper nanoparticles in nitrogen-doped carbonized wood for efficiently enhancing its capacitive performance and electrocatalytic hydrogen evolution, Chemical Engineering Journal. 484 (2024) 149454.

[4] J.-F. Gao, J.-F. Hou, L.-B. Kong. Pure Cu particle obtained by ammonia reduction reaction: A new class of electrodes for hybrid supercapacitors, Journal of Energy Storage. 39 (2021) 102636.

[5] W. Yao, D. Zheng, Z. Li, et al. MXene@ carbonized wood monolithic electrode with hierarchical porous framework for high-performance supercapacitors, Applied Surface Science. 638 (2023) 158130.

[6] F. Wang, J. Y. Cheong, J. Lee, et al. Pyrolysis of Enzymolysis‐Treated Wood: Hierarchically Assembled Porous Carbon Electrode for Advanced Energy Storage Devices, Advanced Functional Materials. 31 (2021) 2101077.

[7] F. Wang, J. Y. Cheong, Q. He, et al. Phosphorus-doped thick carbon electrode for high-energy density and long-life supercapacitors, Chemical Engineering Journal. 414 (2021) 128767.

[8] S. Wang, L. Jiang, J. Hu, et al. Dual-functional CuxO/Cu electrodes for supercapacitors and non-enzymatic glucose sensors fabricated by femtosecond laser enhanced thermal oxidation, Journal of Alloys and Compounds. 815 (2020) 152105.

[9] G. Zhang, Y. Li, R. Zhu, et al. Fabrication of Multi‐Layered Paper‐Based Supercapacitor Anode by Growing Cu(OH)_2_ Nanorods on Oxygen Functional Groups‐Rich Sponge‐Like Carbon Fibers, Small. 20 (2023) 2305136.

[10] Q. Zhang, S. Liu, J. Huang, et al. In situ selective selenization of ZIF-derived CoSe2 nanoparticles on NiMn-layered double hydroxide@CuBr_2_ heterostructures for high performance supercapacitors, Journal of Colloid and Interface Science. 655 (2024) 273-285.

[11] A. Zhang, W. Zheng, Z. Yuan, et al. Hierarchical NiMn-layered double hydroxides@CuO core-shell heterostructure in-situ generated on Cu(OH)_2_ nanorod arrays for high performance supercapacitors, Chemical Engineering Journal. 380 (2020) 122486.

[12] A. Zhang, L. Yue, D. Jia, et al. Cobalt/Nickel Ions-Assisted Synthesis of Laminated CuO Nanospheres Based on Cu(OH)_2_ Nanorod Arrays for High-Performance Supercapacitors, ACS Applied Materials & Interfaces. 12 (2019) 2591-2600.

[13] Y. Zhou, Y. Li, H. Chen, et al. Rational synthesis of Cu_7_S_4_/CoS_2_ hybrid nanorods arrays grown on Cu foam from metal-organic framework templates for high-performance supercapacitors, Journal of Alloys and Compounds. 807 (2019) 151680.

[14] H. Wang, G. Yan, X. Cao, et al. Hierarchical Cu(OH)_2_@MnO_2_ core-shell nanorods array in situ generated on three-dimensional copper foam for high-performance supercapacitors, Journal of Colloid and Interface Science. 563 (2020) 394-404.

[15] J. Ouyang, X. Wang, L. Wang, et al. Construction of a porous carbon skeleton in wood tracheids to enhance charge storage for high-performance supercapacitors, Carbon. 196 (2022) 532-539.

[16] Y. Chen, Y. Yu, X. Zhang, et al. High performance supercapacitors assembled with hierarchical porous carbonized wood electrode prepared through self-activation, Industrial Crops and Products. 181 (2022) 114802.

[17] Z. Yu, Z. Cheng, X. Wang, et al. High area-specific capacitance of Co(OH)_2_/hierarchical nickel/nickel foam supercapacitors and its increase with cycling, Journal of Materials Chemistry A. 5 (2017) 7968-7978.

[18] X. He, Y. Hu, H. Tian, et al. In-situ growth of flexible 3D hollow tubular Cu_2_S nanorods on Cu foam for high electrochemical performance supercapacitor, Journal of Materiomics. 6 (2020) 192-199.

[19] Y. Chen, H. Hou, B. Liu, et al. Wood-derived scaffolds decorating with nickel cobalt phosphate nanosheets and carbon nanotubes used as monolithic electrodes for assembling high-performance asymmetric supercapacitor, Chemical Engineering Journal. 454 (2023) 140453.

[20] D. He, G. Wang, G. Liu, et al. Facile route to achieve mesoporous Cu(OH)_2_ nanorods on copper foam for high-performance supercapacitor electrode, Journal of Alloys and Compounds. 699 (2017) 706-712.

[21] R. Si, H. Luo, J. Pu. Constructing nitrogen/sulfur co-doped hierarchical porous cellulose-based carbon derived from larch via regeneration by dissolution approach for supercapacitor, Industrial Crops and Products. 200 (2023) 116844.

[22] L. Chen, F. Wang, Z. Tian, et al. Wood-Derived High-Mass-Loading MnO_2_ Composite Carbon Electrode Enabling High Energy Density and High-Rate Supercapacitor, Small. 18 (2022) e2201307.

[23] L. L. Shen, X. Dong, W. Wang, et al. Amidoximated‐Wood Derived Carbons as Advanced Self‐Standing Electrodes for Supercapacitor and Water Splitting, Advanced Functional Materials. 34 (2024) 2400964.

[24] F. Wang, L. Chen, S. He, et al. Design of wood-derived anisotropic structural carbon electrode for high-performance supercapacitor, Wood Science and Technology. 56 (2022) 1191-1203.

[25] W. Tian, P. Ren, X. Hou, et al. N-Doped Holey Graphene/Porous Carbon/Cellulose Nanofibers Electrode and Hydrogel Electrolyte for Low-temperature Zinc-ion Hybrid Supercapacitors, Small. (2025) e2411657.

[26] J. Cao, L. Lin, J. Zhang, et al. Biological treatment as a green approach for enhancing electrochemical performance of wood derived carbon based supercapacitor electrodes, Journal of Cleaner Production. 422 (2023) 138659.

[27] L. Shi, J. Ye, H. Lu, et al. Flexible all-solid-state supercapacitors based on boron and nitrogen-doped carbon network anchored on carbon fiber cloth, Chemical Engineering Journal. 410 (2021) 128365.

[28] S. Feng, D. Wang, W. Yang, et al. Carbonized wood loading sustainable tannin used as free-standing electrodes for assembling heavy metal-free supercapacitors, International Journal of Biological Macromolecules. 285 (2025) 138381.

[29] M. Gao, Z. Wang, Z. Liu, et al. 2D Conjugated Metal–Organic Frameworks Embedded with Iodine for High‐Performance Ammonium‐Ion Hybrid Supercapacitors, Advanced Materials. 35 (2023) 2305575.

[30] L. Jin, X. Liu, Z. Wang, et al. Fabrication of Porous Reduced Graphene Oxide Encapsulated Cu(OH)_2_ Core–shell Structured Carbon Fiber-Based Electrodes for High-Performance Flexible Supercapacitors, ACS Applied Materials & Interfaces. 15 (2023) 58517-58528.

[31] G. Zhang, R. Zhu, R. Zhang, et al. 3D hetero-nanostructured electrode constructed on carbon fiber paper with 2D 1T-MoS_2_/1D Cu(OH)_2_ for flexible asymmetric solid-state supercapacitors, Journal of Power Sources. 523 (2022) 231031.

[32] Y. Mo, X. Huang, X. Shi, et al. Zn-doped NiCo-LDH nanoflakes grown in-situ on CuO/Cu as an electrode for coaxial flexible asymmetric supercapacitor, Journal of Alloys and Compounds. 1017 (2025) 179131.

[33] Y. Wang, X. Lin, T. Liu, et al. Wood‐Derived Hierarchically Porous Electrodes for High‐Performance All‐Solid‐State Supercapacitors, Advanced Functional Materials. 28 (2018) 1806207.

[34] K. Jiang, S. Yang, W. Chen, et al. Free-Standing Co(OH)_2_/Prussian Blue Analogue Nanostructured Electrodes for Flexible Na-Ion Supercapacitors with an Ultrawide Potential Window, ACS Applied Nano Materials. 7 (2024) 6650-6658.

[35] Q. Yang, Z. Li, B. Xu. Layered Double Hydroxide with Interlayer Quantum Dots and Laminate Defects for High‐Performance Supercapacitor, Advanced Functional Materials. 33 (2023) 2300149.

[36] Z. Yang, Y. Chen, Q. Tian, et al. CuCo_2_S_4_@MnO_2_ hollow porous heterostructure for high-performance supercapacitors, Journal of Alloys and Compounds. 989 (2024) 174361.

[37] P. Zhou, Y. Ji, B. Zhang, et al. Rational construction of tremella-like CuCo LDH@Ni_3_S_2_ nanocomposites as high-performance supercapacitor electrode materials, Journal of Alloys and Compounds. 1021 (2025) 179586.

[38] P.-P. Yao, A. Lv, L.-D. Shi, et al. Multi-level nanostructures of NiCoP/CNTs/CuO nanowire arrays/Cu foam with excellent electrochemical performance for flexible supercapacitors, Journal of Alloys and Compounds. 1009 (2024) 176847.

[39] X. Yang. ZIF-8 derived Zn, Mn, S and P co-loaded on N doped carbon as efficient electrocatalyst for hydrogen evolution reaction (HER), International Journal of Hydrogen Energy. 47 (2022) 18314-18320.

[40] M. Zheng, K. Shi, Y. Zhao, et al. The novel mushroom-like carbon nanotube top-supported FeNi alloy doped P material with ultra-high activity for OER/HER/ORR, International Journal of Hydrogen Energy. 72 (2024) 1077-1090.

[41] J. Yu, W. Yu, B. Chang, et al. Waste‐yeast biomass as nitrogen/phosphorus sources and carbon template: Environment‐friendly synthesis of N,P‐Mo_2_C nanoparticles on porous carbon matrix for efficient hydrogen evolution, Chinese Chemical Letters. 33 (2022) 3231-3235.

[42] Z. Hou, F. Fan, C. Teng, et al. N, P co-doped NiMoOx three-dimensional nanoflower cluster-structured bifunctional electrocatalyst for efficient overall water splitting, Journal of Alloys and Compounds. 1013 (2025) 178510.

[43] X. Wang, J. Dai, H. Xie, et al. In-situ construction of ultrathin MoP-MoS_2_ heterostructure on N, P and S co-doped hollow carbon spheres as nanoreactor for efficient hydrogen evolution, Chemical Engineering Journal. 438 (2022) 135544.

[44] Z. Ajmal, M. Arif, A. Kumar, et al. Uniformaly distributed Ru nanoparticles over N, P co-doped porous carbon as a highly active trifunctional electrocatalyst, International Journal of Hydrogen Energy. 73 (2024) 768-774.

[45] Y. Lu, C. Yue, Y. Li, et al. Atomically dispersed Ni on Mo_2_C embedded in N, P co-doped carbon derived from polyoxometalate supramolecule for high-efficiency hydrogen evolution electrocatalysis, Applied Catalysis B: Environmental. 296 (2021) 120336.

[46] D. Sun, S. Lin, Y. Yu, et al. One-pot synthesis of N and P Co-doped carbon layer stabilized cobalt-doped MoP 3D porous structure for enhanced overall water splitting, Journal of Alloys and Compounds. 895 (2022) 162595.

[47] Z. Cai, A. Li, W. Zhang, et al. Hierarchical Cu@Co-decorated CuO@Co_3_O_4_ nanostructure on Cu foam as efficient self-supported catalyst for hydrogen evolution reaction, Journal of Alloys and Compounds. 882 (2021) 160749.

[48] S. Chandrasekaran, T. Ma, Z. Hu, et al. Delocalization of d-electrons induced by cation coupling in ultrathin Chevrel-phase NiMo_3_S_4_ nanosheets for efficient electrochemical water splitting, Applied Catalysis B: Environmental. 338 (2023) 123007.

[49] S. Chu, W. Chen, G. Chen, et al. Holey Ni-Cu phosphide nanosheets as a highly efficient and stable electrocatalyst for hydrogen evolution, Applied Catalysis B: Environmental. 243 (2019) 537-545.

[50] N. P. Dileep, P. V. Sarma, R. Prasannachandran, et al. Electrostatically Coupled Nanostructured Co(OH)_2_–MoS_2_ Heterostructures for Enhanced Alkaline Hydrogen Evolution, ACS Applied Nano Materials. 4 (2021) 7206-7212.

[51] X. Du, Y. Ding, H. Su, et al. Effect of cation substitution on the water splitting performance of spinel cobaltite MCo_2_S_4_ (M = Ni, Cu and Co), International Journal of Hydrogen Energy. 45 (2020) 12012-12025.

[52] X. Du, L. Yin, W. Zhang, et al. Synergistic coupling of Ni_3_ZnC_0.7_ decorated with homogeneous multimetal CoNiCuFe nitrogen-codoped carbon matrix as high-entropy catalysts for efficient overall water splitting, Journal of Materials Science & Technology. 135 (2023) 26-33.

[53] L. Fathyunes, G. B. Darband, C. Muilwijk, et al. Studying impact of cobalt content on electrocatalytic activity of Cu-Co-P coating with dendrite-like architectures for hydrogen production, Journal of Environmental Chemical Engineering. 12 (2024) 113657.

[54] M. Wang, M. Li, Y. Zhao, et al. Construction of N-doped carbon frames anchored with Co single atoms and Co nanoparticles as robust electrocatalyst for hydrogen evolution in the entire pH range, Journal of Energy Chemistry. 67 (2022) 147-156.

[55] B. Gao, X. Du, Y. Zhao, et al. Electron strain-driven phase transformation in transition-metal-co doped MoTe_2_ for electrocatalytic hydrogen evolution, Chemical Engineering Journal. 433 (2022) 133768.

[56] C. Gong, L. Zhao, D. Li, et al. In-situ interfacial engineering of Co(OH)_2_/Fe_7_Se_8_ nanosheets to boost electrocatalytic water splitting, Chemical Engineering Journal. 466 (2023) 143124.

[57] R. Jin, J. Huang, G. Chen, et al. Water-sprouted, plasma-enhanced Ni-Co phospho-nitride nanosheets boost electrocatalytic hydrogen and oxygen evolution, Chemical Engineering Journal. 402 (2020) 126257.

[58] X. Li, Z. Zhao, X. Meng, et al. Rapid Joule-heating fabrication of Ce-doped Co/CoO on carbon nanotubes for efficiently electrocatalytic hydrogen production, Applied Surface Science. 669 (2024) 160481.

[59] H. Liu, Z.-S. Wu, L. Huang, et al. Mechanistic insights into interfaces and nitrogen vacancies in cobalt hydroxide/tungsten nitride catalysts to enhance alkaline hydrogen evolution, Journal of Materials Chemistry A. 9 (2021) 11323-11330.

[60] N. Song, S. Hong, M. Xiao, et al. Fabrication of Co(Ni)-P surface bonding states on core–shell Co(OH)_2_@P-NiCo-LDH towards electrocatalytic hydrogen evolution reaction, Journal of Colloid and Interface Science. 582 (2021) 535-542.

[61] H. Su, S. Song, S. Li, et al. High-valent bimetal Ni_3_S_2_/Co_3_S_4_ induced by Cu doping for bifunctional electrocatalytic water splitting, Applied Catalysis B: Environmental. 293 (2021) 120225.

[62] S. Swathi, R. Yuvakkumar, G. Ravi, et al. Nanoplatelets assembled CuCo_2_S_4_/N doped rGO nanocomposites for hydrogen evolution reaction, International Journal of Hydrogen Energy. 65 (2024) 704-716.

[63] G. Wang, J. Huang, G. Chen, et al. In-Situ-Engineered 3D Cu_3_Se_2_@CoSe_2_–NiSe_2_ Nanostructures for Highly Efficient Electrocatalytic Water Splitting, ACS Sustainable Chemistry & Engineering. 8 (2020) 17215-17224.

[64] Y. Wan, W. Wei, S. Ding, et al. A Multi‐Site Synergistic Effect in High‐Entropy Alloy for Efficient Hydrogen Evolution, Advanced Functional Materials. (2024) 2414554.

[65] Y. Wang, B. Wang, S. Xia, et al. Enhanced hydrogen evolution performance of Co/CoP in electrolysis of water by interfacial regulation of crystal plane orientation, International Journal of Hydrogen Energy. 67 (2024) 1106-1116.

[66] Y. Wu, D. Gao, L. Huang, et al. In situ electrochemical construction of Co/CoP crystalline-amorphous hetero-phase catalysts for highly efficient electrocatalytic hydrogen evolution, Chemical Communications. 59 (2023) 2429-2432.

[67] X. Xu, F. Qiao, Y. Liu, et al. Preparation of Cu(OH)_2_/Cu_2_S arrays for enhanced hydrogen evolution reaction, Battery Energy. 3 (2024) 20230060.

[68] J. Yu, Y. Wang, Y. Jing, et al. Improving electrocatalytic hydrogen evolution through Co and Ni single atom sites with synergistic effects anchored on carbon foam as a self-supporting electrode, Chemical Engineering Journal. 484 (2024) 149406.

[69] H. Zhang, X. Du, X. Zhang, et al. Controlled synthesis of NiCoP@NiM LDH (M=Cu, Fe, Co) as efficient hydrogen evolution reaction electrocatalyst, Journal of Alloys and Compounds. 937 (2023) 168412.
